# Gender, BMI and fasting hyperglycaemia influence Monocyte to-HDL ratio (MHR) index in metabolic subjects

**DOI:** 10.1371/journal.pone.0231927

**Published:** 2020-04-28

**Authors:** Stefano Battaglia, Natasha Scialpi, Elsa Berardi, Gianfranco Antonica, Patrizia Suppressa, Francesco Arcangelo Diella, Francesca Colapietro, Roberta Ruggieri, Giuseppe Guglielmini, Alessia Noia, Giusi Graziano, Carlo Sabbà, Marica Cariello

**Affiliations:** 1 Department of Interdisciplinary Medicine, “Aldo Moro” University of Bari, Bari, Italy; 2 Department of Tissues and Organs Transplantation and Cellular Therapies, “Aldo Moro” University of Bari, Bari, Italy; 3 INBB, National Institute for Biostructures and Biosystems, Rome, Italy; Universidade Federal de Goias, BRAZIL

## Abstract

Metabolic Syndrome (MS) is characterized by a low-grade inflammatory state causing an alteration of non-invasive indexes derived from blood count, namely monocyte-to-HDL ratio (MHR), neutrophil-to-lymphocyte ratio (NLR), platelet-to-lymphocyte ratio (PLR), lymphocyte-to-monocyte ratio (LMR). We analyse a population of 771 subjects (394 controls and 377 MS patients) to evaluate the best predictive index of MS. The diagnosis of MS was made according to the 2006 criteria of the International Diabetes Federation (IDF). We performed ROC curve analyses to evaluate the best predictor index of MS. MHR cut-off value was used to classify the population in two different groups and to create the outcome variable of the Recursive Partitioning and Amalgamation (RECPAM) analysis. This method is a tree-structured approach that defines "risk profiles" for each group of dichotomous variables. We showed that MHR index is significantly linked to body mass index (BMI), waist circumference, creatinine, C-reactive protein (CRP), Erythrocyte Sedimentation Rate (ESR). ROC curve defined an MHR cut-off value of 6.4, which was able to identify two patient groups with significant differences in waist circumference, blood pressure, creatinine, estimated glomerular filtration rate and fasting plasma glucose. RECPAM analysis demonstrated that gender, BMI categorization and hyperglycaemia were the most important risk determinants of increased MHR index that can be considered bona fide a useful and easily obtainable tool to suggest the presence of peculiar metabolic features that predict MS.

## Introduction

Metabolic Syndrome (MS) is a systemic condition characterized by a wide spectrum of clinical features as central obesity, hypertension, and impaired glucose and lipid homeostasis [1]. During the last 20 years, the definition of MS has been subjected to several modifications. In 2006, the International Diabetes Federation (IDF) established the following criteria to define the MS[2], i.e. fasting plasma glucose (FPG) ≥100 mg/dL (or drug treatment for elevated glucose); high density lipoprotein (HDL) concentration <40 mg/dL in males or <50 mg/dL in females (or drug treatment for dyslipidaemia); triglycerides (TG) ≥150 mg/dL (or drug treatment for elevated triglycerides); waist circumference (WC) >94 cm in males or >80 cm in females and Systolic Arterial Pressure (SAP) ≥130 mmHg or diastolic blood pressure ≥85 mmHg (or with antihypertensive treatment).

The elevated values of waist circumference and the presence of two or more other criteria leads to the diagnosis of MS. Notably, the IDF definition underlines the crucial importance of waist circumference as a non-invasive method to detect the presence of visceral adipose tissue. Indeed, the accumulation of visceral fat plays a critical role in the pathogenesis of MS and it is commonly considered as the “primary trigger” of MS alterations[3, 4]. Moreover, MS is characterized by a low-grade inflammatory state caused by the increased production of cytokines, chemokines, and adipokines and the abnormal activation of immune cells[4], that collectively contribute to the atherosclerotic plaque formation and Non-Alcoholic Fatty Liver Disease (NAFLD) [3].

The detection of the “chronic inflammatory state” with non-invasive indexes is considered a crucial goal for clinicians. Non-invasive parameters like C-reactive protein (CRP), fibrinogen and pro-inflammatory cytokines like interleukin (IL)-6, IL-10 and tumor necrosis factor-α (TNF-*α*) have been studied to identify the pro-inflammatory state of MS patients[5].

Recently, leukocytes’ subtypes have been considered useful systemic inflammation markers [6] as well as neutrophil-to-lymphocyte ratio (NLR) [7, 8], platelet-to-lymphocyte ratio (PLR) [9, 10], lymphocyte-to-monocyte ratio (LMR) [11, 12]and monocyte-to-HDL ratio (MHR)[13–18] indexes. Furthermore, several studies underlined the link between NLR, PLR, LMR, MHR and cardiovascular diseases, like peripheral arterial occlusive disease, coronary artery diseases (including myocardial infarction), atrial fibrillation and aortic alterations but the association with MS is still not well established [12, 16, 19–21].

Buyukkaya *et al*, showed that NLR was significantly higher in MS patients and it increased with the severity of the disease[8]. In 2016, Akboga *et al*. demonstrated that MS patients presented higher PLR values compared to controls[22]. Vahit *et al*. evaluated the association of LMR and MHR with MS, observing that MHR was higher in MS patients than controls, whereas LMR followed the opposite trend[11]. Furthermore, Uslu *et al*. identified MHR cut-off value of 9.36 as predictive of MS[17].

In the present study, we analysed control and metabolic subjects characterized by chronic comorbidities (diabetes mellitus, hypertension, chronic kidney disease) with the aim of studying the association of NLR, PLR, MHR, LMR indexes with clinical features of MS to evaluate the best predictive index of this pathology.

## Materials and methods

### Study population

**Fig 1:**
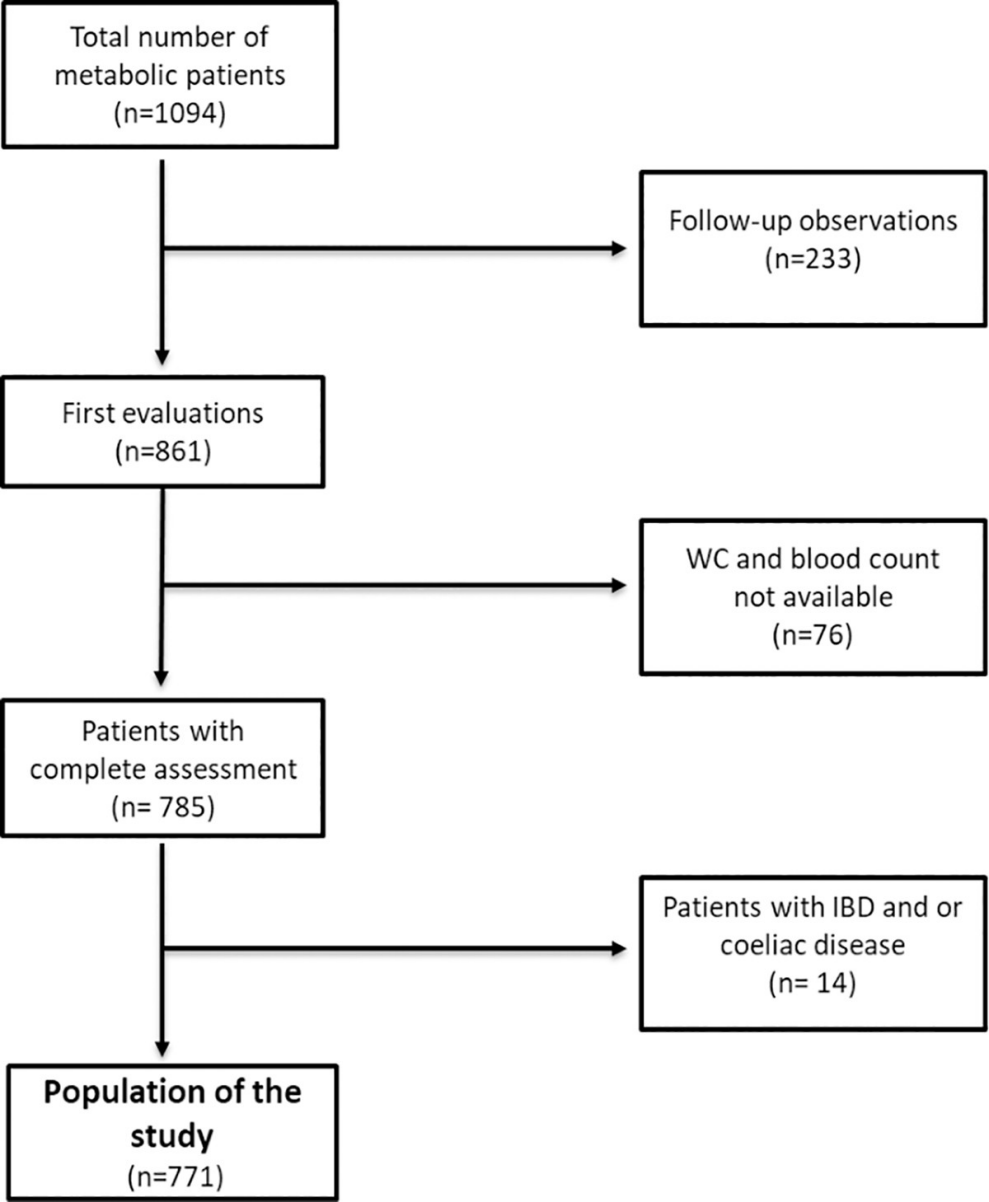
Flow chart of the study population.

Patient recruitments, clinical and biochemical assessment have been consecutively recorded from 2014 to February 2019 in the electronic health register of Metabolic Diseases of Department of Interdisciplinary Medicine, Internal Medicine Division, “Aldo Moro” University of Bari, Italy. This register included 1094 patients affected by metabolic diseases like MS, diabetes mellitus, hypertension, and dyslipidemias. Among the 1094 patients, 861 were first evaluations whereas 233 were follow-up observations that were excluded from the study. Next, we noticed that 64 out of these 861 patients lacked the value of waist circumference, so they were not included in the evaluation. Then, we observed that blood count was not available for 12 patients of this population of 797 patients, thence removed them from the study population reaching the number of 785 patients. The diagnosis of Inflammatory Bowel Disease and/or Celiac disease allowed us to rule out 14 patients more. Indeed, the population under study reached the number of 771, whose data were free from any analysis restriction (Fig 1, S1 Table). Exclusion criteria: renal and hepatic failure, acute heart diseases (cardiac failure, coronary arterial disease, acute arrhythmias), infections, treatment with medications affecting the number of leukocytes. The following chronic conditions were considered as exclusion criteria: secondary hypertension, chronic systemic inflammatory diseases, and neoplastic diseases with recent onset (less than 10 years) and/or under chemotherapy. The population included patients with chronic hypertension (also with a chronic condition of hypertensive cardiopathy), chronic diabetes, chronic renal failure, obesity and chronic gastrointestinal diseases (gastroesophageal reflux, chronic gastritis, irritable bowel syndrome).

In order to calculate the sample size of the study we assumed to detect a difference of at least 3 in the MHR value between patients with metabolic syndrome and controls, a significance level of 0.05 and power of 90% obtaining a total of 172 subjects to enrol. The study protocol was approved by the Ethical Committee of the Azienda Ospedaliero-Universitaria Policlinico di Bari, Italy. All patients gave their written informed consent for the use of clinical data.

### Clinical evaluation and anthropometric measurements

Anthropometric parameters (height, body weight, waist and hip circumference) were measured using standardized procedures. Waist circumference (WC) was measured at the midpoint between the inferior part of the 12^th^ costa and the anterior-superior iliac crests. BMI (body mass index) was calculated as body weight (expressed in kg) divided by squared height expressed in meters (m^2^); conditions of normal weight, overweight and obesity were identified according to BMI values of respectively 20.0–24.9, 25.0–29.9 and 30.0 kg/m^2^. Systolic and diastolic blood pressure were determined after three repeated measurements.

Diabetes Mellitus (DM) and Hypertension were defined according to the criteria accepted by the International community. For DM the criteria were: HbA1c (percentage of glycosylated haemoglobin) ≥6.5%, fasting plasma glucose (FPG) ≥126mg/dL, blood glucose ≥200mg/dL and/or treatment for diabetes. Hypertension was defined as systolic arterial blood pressure (SAP)≥140mmHg, diastolic arterial blood pressure (DAP) ≥90mmHg and/or treatment with antihypertensive agents. To define dyslipidaemia, HDL cut-off was <40 mg/dL in men and <50 mg/dL in women. In addition, the reference value of TG was 150 mg/dL for both genders, whereas a total cholesterol level of ≥200 mg/dl was used for the diagnosis of hypercholesterolemia. The diagnosis of MS was made according to 2006 IDF definition[2]. Thence, the population was classified also considering the presence/absence of each criterion of MS and the assumption of a specific category of lipid-lowering drugs. Notably, lipid-lowering treatments were also divided in two groups: patients under statins (n. 154) and other lipid-lowering drugs. The second group of patients included 10 patients treated with fibrates and 24 under the assumption of omega-3 fatty acids, among whom there were 9 patients that assumed also statins.

### Biochemical measurements

The study population underwent blood tests after 12-hour fasting for the measurement of total cholesterol, Triglycerides, HDL Cholesterol, fasting plasma glucose through enzymatic colorimetric assay (Siemens, Erlangen, Germany). CRP, measured via nephelometry (Siemens, Erlangen, Germany) and Erythrocyte Sedimentation Rate (ESR) were additionally evaluated in subgroups of patients. A complete blood count with the determination of leukocyte’s subpopulations was performed. NLR, PLR, LMR and MHRwere calculated manually and LDL cholesterol concentration was obtained using the Friedewald formula.

### Statistical analysis

Statistical analyses were performed using the R statistical environment, version 3.5.2 (The R Foundation for Statistical Computing; Vienna, Austria). Most variables were > 95% complete and the main relevant exceptions were CRP and ESR, since they accounted for 40% of missing data. Shapiro-Wilk test and graphical evaluations of each variable were performed to demonstrate the correspondence with the normal distribution. Student t-test or Mann-Whitney *U* test were performed to assess comparisons among two groups in terms of continuous variables, while differences between more than two groups were studied through one-way analysis of variance (ANOVA) followed by Tukey post-hoc test, where necessary. Pearson χ^2^ test was used for comparisons in terms of categorial variables.

The receiver–operating characteristic (ROC) curves were used to determine optimum cut-off levels of non-invasive indexes[23]. P values lower than 0.05 were regarded as significant.

To assess the interplay among some clinical and demographic variables and to identify internally homogeneous subgroups of patients with different risks of exceeding MHR cut-off, we used the Recursive Partitioning and Amalgamation (RECPAM) method [24, 25] in a multivariate logistic fashion. RECPAM is a tree-structured approach that reproduces the human cognitive process. RECPAM offers a simple presentation of the information about a dichotomous response variable contained in a set of predictors by grouping observations and determining "risk profiles" for each group. Based on available information it selects only those variables with significant information content, and provides a tree structure where each patient is assigned to a single group. Starting from the whole data set, represented by the root node, RECPAM produces by recursively splitting a tree as a clear description of complex interactions among clinical, socioeconomic and other prognostic factors. The selected variable at each node/step (and its best split) is the one with the highest discriminating power while adjusting for all the other variables explored. The recursive partitioning is regulated by stopping rules: the process is stopped only when a user defined minimum node size or a minimum node event number is reached. The final leaves, namely RECPAM classes, are obtained by a pruning and amalgamation sequence of the initial large tree. In particular, the RECPAM classes correspond to subgroups of patients associated with a different risk in terms of outcome variable (in our study the risk of exceeding MHR cut-off). For the elaboration of RECPAM approach, we included in the analysis all the following categorical variables: Gender, Body Mass Index Categories, Waist Circumference Categories, Hypertension, Criterion hyperglycaemia of metabolic syndrome, Criterion TG of metabolic syndrome and age as the exclusive continuous variable. The minimal number of events and the minimal node size (stopping rules) were set to 15 and 60, respectively. The final honest tree was chosen by applying the AIC principle and the graphical “elbow” rule. These approaches were adopted to overcome the model overfitting and to ensure a greater generalizability of the results to future data. The risk, for each subgroup of patients, was expressed as Adjusted Odds Ratio (AdjOR) and the results were summarized in the canonical RECPAM graph where splitting variables were written between different branches and the specific level that brings to the dichotomization was indicated on the relative branch. The subgroups of patients were represented with circles and squares while the numbers inside represented patients over (top) and under (bottom) the MHR cut-off value. The class placed at the extreme right was considered as the reference category (AdjOR = 1.0). The RECPAM analysis was performed using SAS Language (Release 9.4; SAS Institute, Cary, NC) macro routine.

## Results

### Clinical characterization of the study population

Fig 1 represents a flow diagram that describes the process of selection of the population. We show the baseline characteristics of our population in S2 Table. 377 MS patients were compared to 394 controls (Table 1). MS prevalence increases with age in a gender-specific manner: it is higher in men under 50 years and it reverses over 50 years[26]. In line with this, MS patients were older than controls and they were mainly males. Compared to healthy subjects, MS patients exhibited increased BMI, waist circumferences, blood pressure, hypertriglyceridemia, decreased HDL and increased levels of inflammation markers (CRP, ESR, WBC, neutrophils, lymphocytes). Considering non-invasive indexes, MS patients presented increased values of MHR and NLR, but decreased values of PLR and LMR, compared to controls.

**Table 1:** Characterization of the study population.

Variabile	Controls	Metabolic Syndrome	p-value
	**394**	**377**	**-**
**Age** (years)*	51.12±14.90	61.80±12.08	<0.0001
**BMI (**kg/*m*^2^)*	25.25±4.80	30.81±5.66	<0.0001
**Waist circ**. (cm)*	91.97±12.67	107.27±13.28	<0.0001
**SAP** (mmHg)*	121.34±15.98	135.15±17.64	<0.0001
**DAP** (mmHg)*	76.53±9.25	81.17±10.82	<0.0001
**Creat**(mg/dL)*	0.79±0.16	0.84±0.22	0.0003
**eGFR** (mL/min)*	96.24±16.15	87.10±18.28	<0.0001
**Fasting Plasma Glucose** (mg/dL)**	85.00[13.00]	108.48[38.25]	<0.0001
**Tot.chol** (mg/dL)*	187.34±36.41	177.47±49.25	0.001
**HDL.chol** (mg/dL)*	60.24±14.79	46.11±12.94	<0.0001
**LDL. chol** (mg/dL)*	108.60±32.59	98.59±35.83	0.0001
**TG** (mg/dL)**	85.00[46]	139.00[96.00]	<0.0001
**CRP** (mg/L)**	2.80[0.10]	2.90[1.9]	<0.0001
**ESR** (mm/H)**	11.00[11.5]	15.50[18]	<0.0001
**WBC** (x10^3^/*L*)*	6.10±1.79	7.05±1.92	<0.0001
**neut.count** (x10^3^/*L*)*	3.54±1.36	4.18±1.44	<0.0001
**lymph.count** (x10^3^/*L*)*	1.98±0.61	2.18±0.83	0.0001
**mon.count** (x10^3^/*L*)*	0.38±0.16	0.44±0.13	<0.0001
MHR*	6.80±3.27	10.41±4.75	<0.0001
NLR*	1.88±0.76	2.06±0.79	0.002
PLR*	130.26±45.25	120.46±49.16	0.004
LMR*	5.55±1.78	5.17±1.95	0.005
**Gender**			
**Male**	173 (43.90)	218 (57.82)	0.0001
**Female**	221 (56.09)	159 (42.17)	
**Smoke**			
**Non smokers**	253(65.00)	250 (67.2)	0.54
**Smokers**	136 (35.0)	122 (32.8)	
**Statin treatment**			
**not in treatment**	340 (86.73)	275 (72.94)	<0.0001
**in treatment**	52 (13.26)	102 (27.05)	
**Other Lipid-Lowering Medication**			
**not in treatment**	390 (98.98)	347 (92.04)	<0.0001
**in treatment**	4 (1.02)	30 (7.96)	

Values are expressed as mean ±Standard Deviation or median [IQR] respectively for normal (*) and non-normal (**) distributed numeric variables, and with n (%) for categorical ones. Each item was compared among the 2 groups using t-test or Mann-Whitney’s U test for quantitative variable and Pearson χ^2^ test for categorial ones. A level of significance of P < 0.05 (two-sided) was used to compare the study groups. **Abbreviations: BMI,** body mass index**; Waist circ**., waist circumference; **SAP,** systolic arterial pression; **DAP,** diastolic arterial pression; **Creat,** creatinine; **eGFR,** *estimated glomerular filtration rate;* **CRP** C-reactive protein; **ESR,** Erythrocyte Sedimentation Rate; **Tot.chol,** total cholesterol; **HDL.chol,** High-density lipoprotein cholesterol; **LDL. chol,** Low-density lipoprotein; **TG**, Triglycerides; **WBC,** white blood cells count; **neut.count**, neutrophils; **lymph.count**, lymphocytes; **mon.count,** monocytes; **MHR,** monocyte to high-density lipoprotein cholesterol ratio**; NLR,** neutrophil to lymphocyte ratio; **PLR,** platelet to lymphocyte ratio; **LMR,** lymphocyte to monocyte ratio.

#### ROC curve analysis of non-invasive indexes to evaluate the best predictor for MS

**Fig 2:**
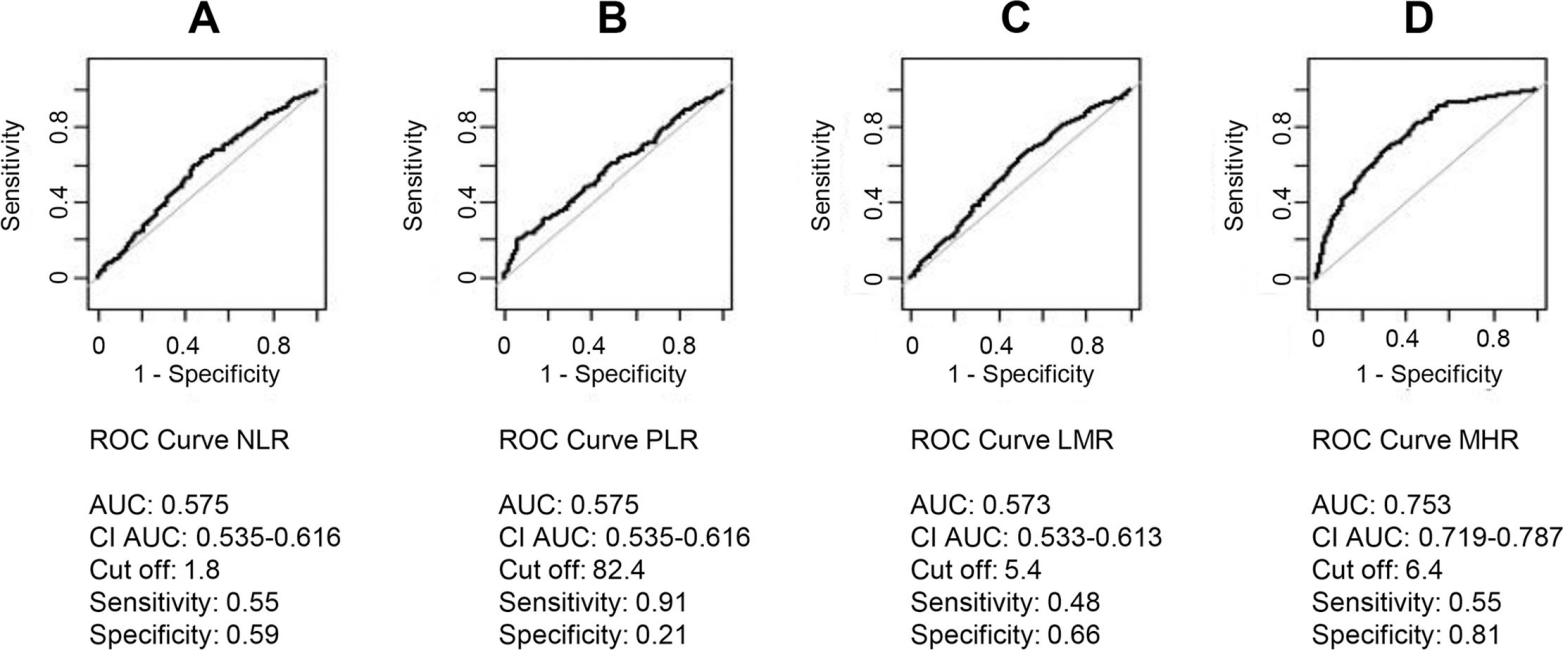
ROC curves of the (A) neutrophil-to-lymphocyte ratio (NLR), (B) platelet-to-lymphocyte ratio (PLR), (C) lymphocyte-to-monocyte ratio (LMR) and (D) monocyte to high-density lipoprotein cholesterol ratio (MHR). The graphs indicated cut off values with respective sensitivity and specificity levels and area under curve (AUC) values with 95% confidence intervals.

**Fig 3:**
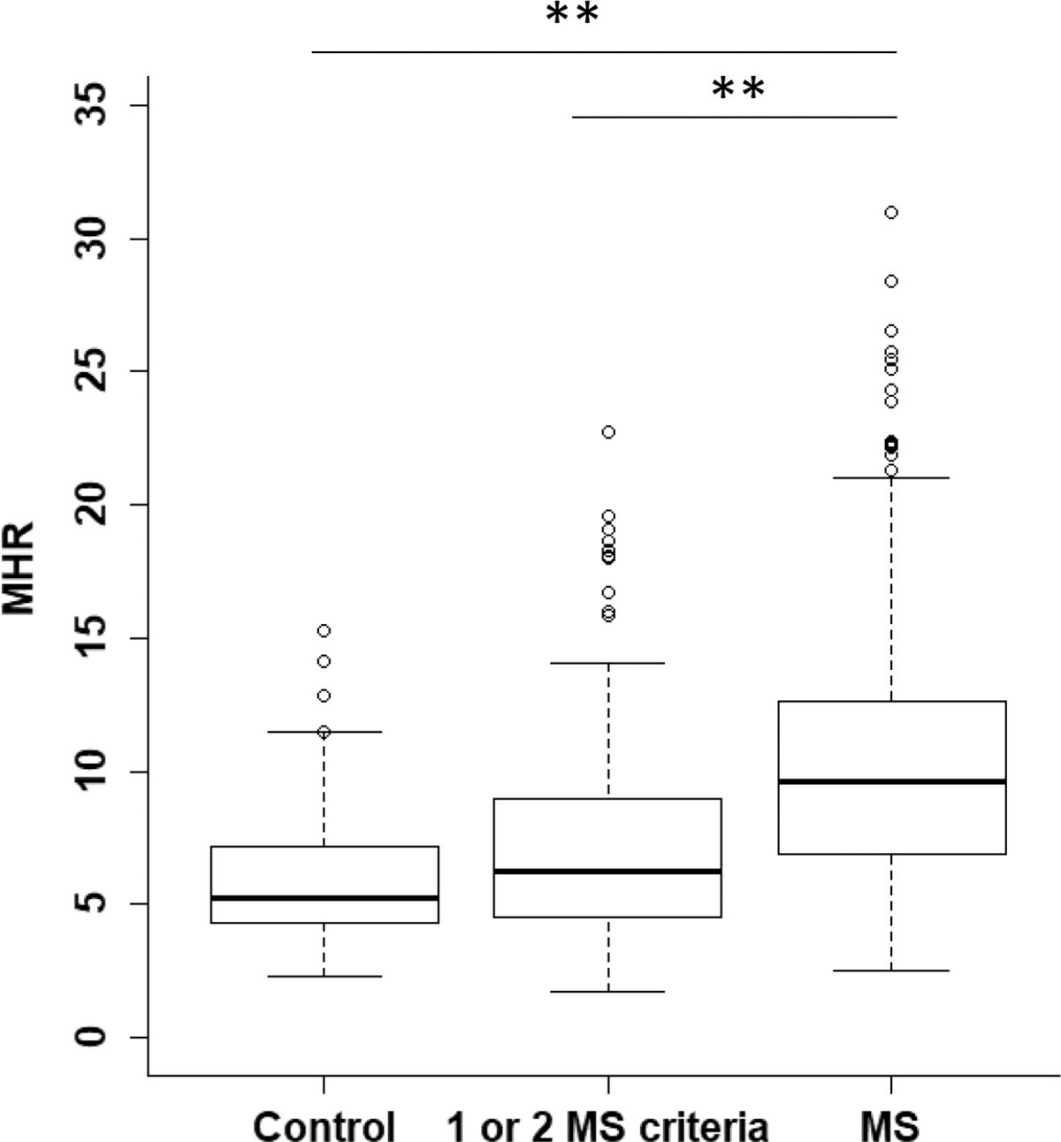
Correlation of monocyte to HDL ratio (MHR) with MS criteria. Box-plot showing the distribution of MHR values in control, patients with 1 or 2 MS criteria and MS patients. The horizontal lines represent the limits of quartiles, whereas the thicker central one indicates the median. Dots above the upper limits shows outliers. ANOVA test detected a significant difference among groups (p-value<0.001) ** p-value<0.001 for Tukey post-hoc test.

In order to find the best predictive index of MS, we performed ROC curve analyses of MHR, NLR, PLR and LMR (Fig 2). The ROC curve of NLR, PLR and LMR presented a low area under the curve (AUC) proving that these indexes do are not able to discriminate MS patients. The ROC curve of MHR was characterized by high specificity and good sensibility (81.7% and 55.8%, respectively) identifying MHR as the best predictive index of MS, with a cut-off value of 6.4 AUC: 0.752, 95% confidence interval: 0.711–0.786; Fig 2). Furthermore, we observed that MHR index significantly increased with the number of MS criteria (ANOVA p-value<0.001 and Tukey post-hoc p-value<0.001; Fig 3)

### Association of MHR index with clinical features of MS patients

The cut-off value obtained by the ROC curve of MHR was used to identify two subgroups of the population (under-/over- cut off) (Table 2). In patients with MHR index over the cut-off, blood pressure, fasting plasma glycemia, TG, chronic inflammation parameters (ESR and CRP) and blood count values were significantly increased compared to patients under cut-off (Table 2). Interestingly, patients with MHR index over the cut-off showed reduced total cholesterol and LDL levels, whereas no difference was found in lipid-lowering treatments. Moreover, MHR index over cut-off value is associated with waist circumference, BMI and MS criteria (Table 2).

**Table 2:** Comparison of clinical variables according to MHR cut-off.

Clinical variable	Under Cut-Off	Over Cut-Off	p-value
	**289**	**482**	
**Age** (years) *	54.66±13.81	57.37±14.97	0.02
**BMI (**kg/*m*^2^)*	25.26±4.79	29.60±5.96	<0.0001
**Waist circ**. (cm)*	92.11±12.63	103.68±14.81	<0.0001
**SAP** (mmHg)*	123.59±16.55	130.81±18.57	<0.0001
**DAP** (mmHg)*	77.50±9.96	79.58±10.45	0.006
**Creat** (mg/dL)*	0.76±0.16	0.85±0.20	<0.0001
**eGFR** (mL/min)*	93.75±15.81	90.58±19.24	0.01
**Fasting Plasma Glucose** (mg/dL)**	87.00[17.00]	98.00[35.00]	<0.0001
**tot.chol** (mg/dL)*	191.66±39.38	177.03±44.82	<0.0001
**HDL.chol** (mg/dL)*	65.90±14.15	46.08±11.08	<0.0001
**LDL. chol** (mg/dL)*	103.00±41.00	100.00±49.75	0.08
**TG** (mg/dL)**	82.00[47.00]	124.00[80.00]	<0.0001
**CRP** (mg/L)**	2.80[0.10]	2.90[1.17]	0.0003
**ESR** (mm/H)**	12.00[11.00]	13.50[16.00]	0.0007
**WBC** (x10^3^/*L*)*	5.30±1.18	7.32±1.87	<0.0001
**neut.count** (x10^3^/*L*)*	3.06±0.96	4.33±1.46	<0.0001
**mon.count** (x10^3^/*L*)*	0.30±0.07	0.47±0.13	<0.0001
**lymph.count** (x10^3^/*L*)*	1.76±0.45	2.26±0.80	<0.0001
**Metabolic Syndrome**			
**Metabolic Syndrome**	69 (23.90)	308 (63.90)	<0.0001
**Controls**	220 (76.10)	174 (36.10)	
**Gender**			
**Male**	84 (29.06)	307 (63.69)	<0.0001
**Female**	205 (70.93)	175 (36.30)	
**Smoke**			
**Non smokers**	214 (42.50)	289 (57.50)	<0.0001
**Smokers**	70 (27.10)	188 (72.90)	
**Body Mass Index**			
**Normal Weight**	151 (52.24)	102 (21.16)	<0.0001
**Overweight**	96 (33.21)	195 (40.45)	
**Obesity**	42 (14.53)	185 (38.38)	
**Waist Circumference**			
**<94 cm (males), <80 cm (females)**	89 (30.79)	88 (18.25)	<0.0001
**>94 cm (males), >80 cm (females)**	200 (69.20)	394 (81.74)	
**Hypertension**			
**controls**	173 (59.86)	163 (33.81)	<0.0001
**diagnosis of hypertension**	116 (40.13)	319 (66.18)	
**Hyperglycemia**			
**Absence**	193 (66.78)	182 (37.75)	<0.0001
**Presence**	96 (33.21)	300 (62.24)	
**Low HDL level**			
**Absence**	264 (91.34)	269 (55.80)	<0.0001
**Presence**	25 (8.65)	213 (44.19)	
**Triglyceridemia**			
**Absence**	260 (89.96)	328 (68.04)	<0.0001
**Presence**	29 (10.03)	154 (31.95)	
**Statin treatment**			
**Not in treatment**	236 (82.22)	379 (78.63)	0.22
**In treatment**	51 (17.77)	103 (21.39)	
**Other Lipid-Lowering Medication**			
**Not in treatment**	280 (96.88)	457 (94.81)	0.17

Values are expressed as mean ±Standard Deviation or median [IQR] respectively for normal (*) and non-normal (**) distributed numeric variables, and with n (%) for categorical ones. Each item was compared among the 2 groups using Student t-test or Mann-Whitney’s U test for quantitative variable and Pearson χ^2^ test for categorial ones. A level of significance of p < 0.05 (two-sided) was used to compare the study groups. **Abbreviations: BMI,** body mass index**; Waist circ**., waist circumference; **SAP,** systolic arterial pression; **DAP,** diastolic arterial pression; **Creat,** creatinine; **eGFR,** *estimated glomerular filtration rate;* **CRP** (C-reactive protein; **ESR,** Erythrocyte Sedimentation Rate; **Tot.chol,** total cholesterol; **HDL.chol,** High-density lipoprotein cholesterol; **LDL.chol,** Low-density lipoprotein; **TG**, Triglycerides; **WBC,** white blood cells count; **neut.count**, neutrophils; **lymph.count**, lymphocytes; **mon.count,** monocytes; **MHR,** monocyte to high-density lipoprotein cholesterol ratio.

**Fig 4:**
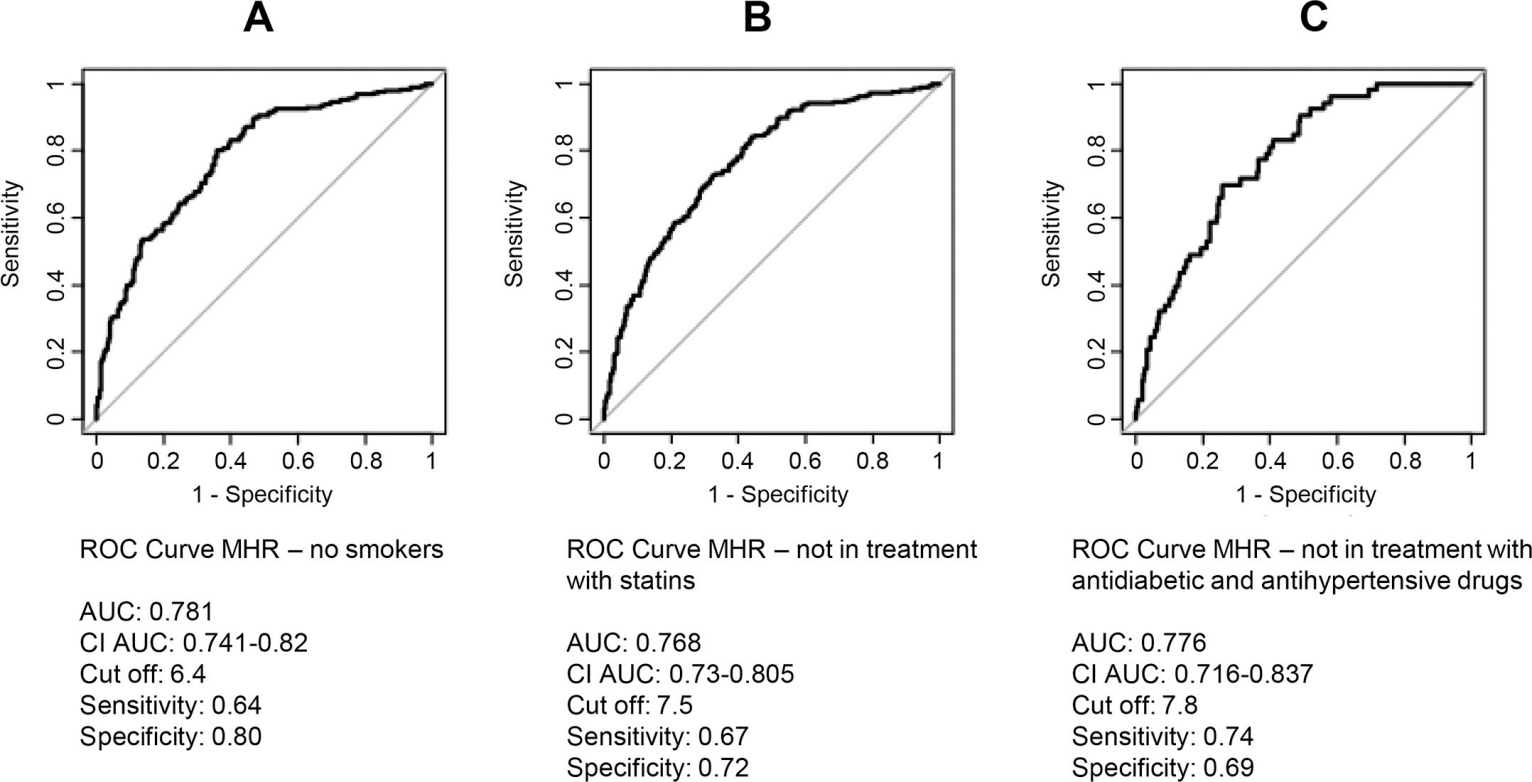
ROC curve of MHR excluding (A) smokers, (B) patients in treatment with statins, (C) patients in treatment with antidiabetic and antihypertensive drugs. The graphs indicated cut off values with sensitivity and specificity levels and area under curve (AUC) values with 95% confidence intervals.

In order to evaluate the influence of smoking habit, statins, anti-hypertensive and anti-diabetic treatments on MHR index we performed ROC curve analyses excluding smokers, patients in treatment with statins and patients in treatment with anti-hypertensive and anti-diabetic drugs (Fig 4A–4C). The ROC curve of MHR excluding smokers showed the same cut-off value and quite similar specificity and sensitivity compared to the analysis based on the complete dataset of patients (Fig 4A). The ROC curve of MHR excluding patients in treatment with statins presented a higher cut-off value but quite similar AUC compared to the ROC curve based on the complete dataset of patients (Fig 4B). Finally, we excluded from our analysis patients in treatment with antidiabetic and antihypertensive drugs leading to a strong sample size reduction (326 patients). The new MHR ROC curve displayed a higher cut-off value and a slight improvement of AUC and sensitivity but a worsen specificity than the ROC curve based on the complete dataset of patients (Fig 4C).

#### RECPAM analysis reveals that Gender, BMI and fasting hyperglycaemia influence MHR index

**Fig 5:**
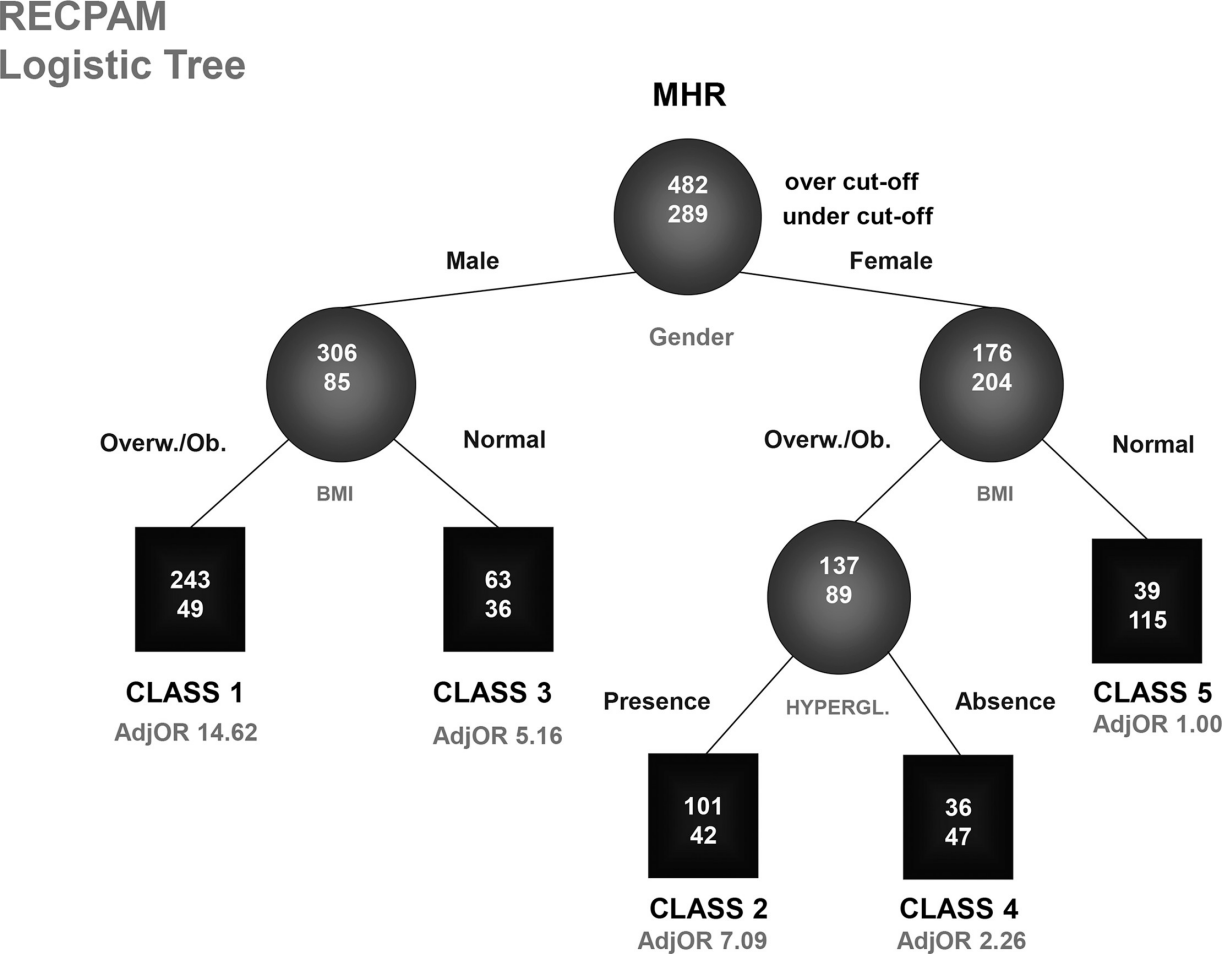
RECPAM logistic tree. Recursive Partitioning and Amalgamation analysis (RECPAM) leads to the identification of patient classes of risk to exceed MHR cut-off. Splitting variables are written between different branches and the specific level that brings to the dichotomization is indicated on the relative branch. Circles and squares indicate subgroups of patients. Numbers inside circles and squares represent patients over (top) and under (bottom) the MHR cut-off value. Adjusted odds ratio (AdjOR) is written below each class. The class with the lowest likelihood to present MHR over cut-off is placed at the extreme right (class 5) and it is considered the reference category (AdjOR = 1.0).

To confirm the role of MHR index as MS predictor, we created a logistic classification tree with the RECPAM analysis (Fig 5). The method identified 5 patient final classes at different risk of exceeding MHR cut-off. The risk was estimated with respect to the reference class 5 and was expressed as Adjusted Odds Ratio (AdjOR). RECPAM analysis categorized BMI in only two subgroups of risk (sharing the subpopulations of overweight and obese in a unique category). The most relevant parameter that influenced the risk of exceeding MHR cut-off for MS patients was gender. Both genders were subdivided according to BMI categories, and we observed that overweight/obese males belonged to higher risk categories (Class 1, AdjOR 14.62), whereas overweight/obese females were furtherly classified according to the presence/absence of hyperglycemia; in particular, women with hyperglycemia had a higher risk (Class 2, AdjOR 7.09) than men with a normal weight (Class 3, AdjOR6.25). Overweight/obese women with a normal glycemic status belonged to Class 4 (AdjOR2.26) and women with normal weight were included in the reference Class 5 (AdjOR1.00). Indeed, the clinical usefulness of MHR index must be evaluated according to gender, BMI and glycemic status; for example, a woman with MHR index over cut-off displays a higher probability to be overweight/obese with hyperglycemia/diabetes.

## Discussion

MS is a condition characterized by several risk factors including central obesity, dyslipidaemia, hypertension, hyperglycaemia and low-grade inflammatory state. Several studies highlighted the involvement of inflammation in MS and atherosclerosis development. In this scenario, the analysis of parameters which can be easily measured from peripheral complete blood count such as PLR, NLR, LMR, and MHR could be useful to evaluate “chronic inflammatory state” in MS patients.

In the present work, we studied the relevance of four non-invasive indexes MHR, PLR, NLR and LMR in a cohort of 771 subjects. By ROC curve analyses we obtained cut-off predictive values of MS, demonstrating that MHR is the best predictor of this pathological condition. Particularly, we identified a cut-off value of 6.4 that is able to discriminate two groups of patients with different metabolic profiles. Furthermore, we showed that gender, BMI categorization and presence/absence of hyperglycaemia are the most important variables influencing MHR index. This index can be considered a simple, cost-effective and useful tool that is able to suggest some important clinical and metabolic features of MS patients.

We observed that MHR index is influenced by gender, in particular overweight/obese males showed a higher risk compare to controls. On the other hand, overweight/obese female patients presented a higher risk when hyperglycaemic. This variable is relevant considering that in MS definition abdominal obesity is classified according to gender. Recently, Laxy *et al*, on pooled data from 41459 patients, demonstrated importance of gender to define BMI cut-off in order to characterize the adipose tissue structure and to predict an “healthy” lifestyle [27]. This can be due to the role of sex hormones to determine body fat distribution, which has an important pathogenic role in MS [28]. Visceral adipocytes can be considered as endocrine cells that produce cytokines, adipokines etc. which control glucose metabolism, appetite, immunological and inflammatory responses, angiogenesis, blood pressure and reproductive function. Furthermore, hyperglycemia can be considered as an important factor of the systemic alterations linked to MS, including the pro-inflammatory state.

HDL cholesterol exerts significant anti-inflammatory effects in the bloodstream through the inhibition of adhesion molecules and chemo-attractive factors for leukocytes, the suppression of monocyte’s activities and their differentiation into macrophages. Moreover, HDL plays an important “protective role” during the formation of atherosclerotic plaque, contributing to reverse cholesterol transport (RCT)[29, 30]. Several therapeutic approaches have been developed in order to maintain a proper level of HDL cholesterol. Statins are drugs able to determine a substantial reduction of LDL concentration [31]. Similarly, fibrates can lead to a moderate increase of HDL-C levels ranging from 5 to 15% [32, 33]. Krysiak *et al*. compared the effect of bezafibrate and omega-3 fatty acids in a group of subjects with isolated hypertriglyceridemia, showing that the first treatment was able to reduce monocyte cytokine release [33]. Furthermore, it has been observed that simvastatin and fenofibrate exhibit a similar inhibitory effect on the secretory function of human monocytes and lymphocytes[34]. Although these approaches exert positive effect on HDL and inflammation, they may condition the non-invasive index considered. Here, for the first time, we demonstrated that in our population, lipid-lower treatments do not influence MHR index values.

Several studies analysed the role of MHR index in the prediction of many different pathological conditions [16]. MHR has been reported to be a prognostic marker in cardiovascular diseases, like primary hypertension, asymptomatic abdominal aortic aneurysm, isolated coronary artery ectasia, coronary atherosclerosis in patients with stable coronary artery disease, acute coronary syndrome [12, 16]. Moreover, this index correlates with major adverse effects in patients after percutaneous coronary intervention for a STEMI or coronary artery bypass grafting[14]. In addition, high MHR values were found in patients with infective endocarditis and normal left ventricular ejection fraction, reflecting its value as a predictor of systemic inflammation [15].

Notably, two studies investigated the association with MHR and MS using the diagnosis of MS according to the National Cholesterol Education Program Adult Treatment Panel III (ATP III) criteria. In 2017, Vahit *et al*.[11] considered a population of 762 patients (371 with MS and 392 controls) and Uslu *et al*.[17] analysed 147 MS patients and 134 healthy controls matched for age and gender, but patients in treatment with lipid-lowering drugs were excluded. This study identified a correlation of MHR index with the severity of MS and MHR cut-off value of 9.39. Differently, our study adopted the IDF definition of MS, which differs from ATP III about the criterion of abdominal obesity. In particular, ATPIII uses waist circumference values greater than IDF [2, 35]. Furthermore, we did not exclude patients in treatment with lipid-lowering drugs and we evaluate the capacity of gender, BMI and hyperglycaemia to influence MHR index.

The main limitation of our study is the retrospective nature of the project, that does not allow an analysis of available data and clinical conditions of patients in the present day. The evaluation of the role of lipid-lowering treatments is limited by the number of patients using these drugs as well as statins. Furthermore, it is important to underline that the RECPAM analysis was only run on the data at hand. We used the pruning step to avoid overfitting results. We have not performed any external validation and we have not verified on a separate testing data how well the model works to determine the generalizability of our results to future or similar settings.

In conclusion, we found that MHR index seems to be a “surrogate marker” that reveals the complex interplay between pro-inflammatory/pro-atherogenic factors (monocytes, cytokines, etc.), alteration of lipid metabolism and anti-inflammatory/atheroprotective factors (HDL cholesterol) in MS patients. The high values of MHR index in MS patients and its association with gender, BMI and hyperglycaemia suggested that MHR might be a useful marker to suggest the presence of peculiar metabolic features and to bona fide predict MS.

## Supporting information

S1 Table Database of the study population. (DOCX)

S2 Table Baseline characteristics of the population. Values are expressed as mean ±Standard Deviation or median ± [IQR] respectively for normal (*) and non-normal (**) distributed numeric variables, and with n (%) for categorical ones. **Abbreviations: Number Obs.,** number of available data**; St.Dev,** standard deviation**; IQR,** interquartilic range range, **NA,** number of not available data**; BMI,** body mass index**; Waist circ**., waist circumference; **SAP,** systolic arterial pression; **DAP,** diastolic arterial pression; **Creat,** creatinine; **eGFR,** *estimated glomerular filtration rate;* **CRP**,C-reactive protein; **ESR,** Erythrocyte Sedimentation Rate; **Tot.chol,** total cholesterol; **HDL.chol,** High-density lipoprotein cholesterol; **LDL. chol,** Low-density lipoprotein; **TG**, Triglycerides; **WBC,** white blood cells count; **neut.count**, neutrophils; **lymph.count**, lymphocytes; **mon.count,** monocytes; **MHR,** monocyte to high-density lipoprotein cholesterol ratio; **NLR,** neutrophil to lymphocyte ratio; **PLR,** platelet to lymphocyte ratio; **LMR,** lymphocyte to monocyte ratio.
(DOCX)
