# Supplementary material for: Gender, BMI and fasting hyperglycaemia influence Monocyte to-HDL ratio (MHR) index in metabolic subjects
Source: PLoS One. 2020 Apr 28;15(4):e0231927. doi: 10.1371/journal.pone.0231927 (PMC7188261; doi:10.1371/journal.pone.0231927)
Supplement: S1 Table — (DOCX) [file pone.0231927.s001.docx]

**Table S1. Database of the study population**

| **Patients ID** | **Gender** | **BMI** | **Fasting Plasma Glucose** | **MHR index** |
| --- | --- | --- | --- | --- |
| PATIENT1 | 1 | 20,70 | 87 | 3,84 |
| PATIENT2 | 0 | 25,40 | 96 | 5,44 |
| PATIENT3 | 1 | 38,57 | 103 | 6,92 |
| PATIENT4 | 0 | 28,08 | 83 | 5,01 |
| PATIENT5 | 0 | 26,30 | 134 | 11,66 |
| PATIENT6 | 0 | 25,40 | 121 | 7,04 |
| PATIENT7 | 1 | 24,06 | 77 | 6,29 |
| PATIENT8 | 1 | 22,48 | 82 | 4,98 |
| PATIENT9 | 1 | 23,50 | 91 | 3,21 |
| PATIENT10 | 1 | 36,51 | 82 | 11,24 |
| PATIENT11 | 1 | 21,77 | 79 | 4,94 |
| PATIENT12 | 1 | 24,61 | 84 | 4,51 |
| PATIENT13 | 1 | 40,40 | 89 | 5,04 |
| PATIENT14 | 0 | 23,41 | 138 | 20,19 |
| PATIENT15 | 1 | 25,27 | 103 | 3,03 |
| PATIENT16 | 0 | 27,55 | 83 | 10,27 |
| PATIENT17 | 0 | 28,52 | 84 | 7,58 |
| PATIENT18 | 0 | 24,38 | 97 | 10,20 |
| PATIENT19 | 1 | 27,16 | 81 | 6,23 |
| PATIENT20 | 1 | 34,96 | 160 | 9,38 |
| PATIENT21 | 1 | 29,55 | 261 | 10,36 |
| PATIENT22 | 1 | 25,51 | 81 | 3,32 |
| PATIENT23 | 1 | 30,85 | 86 | 13,55 |
| PATIENT24 | 0 | 29,86 | 94 | 6,46 |
| PATIENT25 | 0 | 35,80 | 92 | 15,66 |
| PATIENT26 | 1 | 21,30 | 70 | 7,42 |
| PATIENT27 | 0 | 44,29 | 91 | 17,69 |
| PATIENT28 | 1 | 25,00 | 83 | 4,73 |
| PATIENT29 | 1 | 29,41 | 109 | 9,78 |
| PATIENT30 | 1 | 17,85 | 72 | 4,17 |
| PATIENT31 | 1 | 20,31 | 75 | 10,49 |
| PATIENT32 | 1 | 21,10 | 86 | 4,33 |
| PATIENT33 | 0 | 37,09 | 138 | 8,78 |
| PATIENT34 | 1 | 30,86 | 80 | 28,36 |
| PATIENT35 | 1 | 26,22 | 75 | 5,30 |
| PATIENT36 | 1 | 34,88 | 84 | 13,23 |
| PATIENT37 | 0 | 28,89 | 116 | 15,44 |
| PATIENT38 | 1 | 36,13 | 90 | 4,44 |
| PATIENT39 | 1 | 24,89 | 121 | 10,58 |
| PATIENT40 | 0 | 40,74 | 122 | 10,02 |
| PATIENT41 | 0 | 26,81 | 88 | 10,93 |
| PATIENT42 | 0 | 32,04 | 84 | 17,86 |
| PATIENT43 | 1 | 25,78 | 92 | 3,20 |
| PATIENT44 | 0 | 19,84 | 78 | 2,27 |
| PATIENT45 | 1 | 27,61 | 93 | 3,16 |
| PATIENT46 | 1 | 25,71 | 83 | 6,22 |
| PATIENT47 | 1 | 36,72 | 199 | 8,76 |
| PATIENT48 | 0 | 25,25 | 87 | 21,27 |
| PATIENT49 | 0 | 25,38 | 104 | 5,94 |
| PATIENT50 | 1 | 14,87 | 76 | 4,74 |
| PATIENT51 | 1 | 23,44 | 74 | 6,63 |
| PATIENT52 | 1 | 32,81 | 136 | 16,11 |
| PATIENT53 | 1 | 25,00 | 85 | 2,39 |
| PATIENT54 | 1 | 40,44 | 90 | 7,09 |
| PATIENT55 | 0 | 28,09 | 99 | 7,47 |
| PATIENT56 | 1 | 29,02 | 72 | 6,08 |
| PATIENT57 | 0 | 24,00 | 95 | 4,61 |
| PATIENT58 | 0 | 33,08 | 106 | 9,00 |
| PATIENT59 | 1 | 22,60 | 80 | 3,20 |
| PATIENT60 | 0 | 29,71 | 94 | 10,69 |
| PATIENT61 | 0 | 27,73 | 181 | 10,67 |
| PATIENT62 | 0 | 25,16 | 83 | 14,86 |
| PATIENT63 | 0 | 23,80 | 84 | 6,77 |
| PATIENT64 | 0 | 34,81 | 77 | 9,94 |
| PATIENT65 | 0 | 24,69 | 79 | 4,76 |
| PATIENT66 | 1 | 21,45 | 88 | 5,26 |
| PATIENT67 | 0 | 27,69 | 135 | 10,66 |
| PATIENT68 | 0 | 28,08 | 143 | 9,11 |
| PATIENT69 | 1 | 32,88 | 87 | 4,59 |
| PATIENT70 | 1 | 29,52 | 215 | 11,88 |
| PATIENT71 | 0 | 20,09 | 83 | 7,74 |
| PATIENT72 | 0 | 28,41 | 108 | 6,57 |
| PATIENT73 | 0 | 27,01 | 94 | 3,87 |
| PATIENT74 | 1 | 26,63 | 115 | 3,86 |
| PATIENT75 | 1 | 19,10 | 86 | 5,78 |
| PATIENT76 | 1 | 16,99 | 68 | 11,15 |
| PATIENT77 | 0 | 24,22 | 75 | 8,99 |
| PATIENT78 | 1 | 34,82 | 110 | 9,46 |
| PATIENT79 | 0 | 44,60 | 94 | 12,41 |
| PATIENT80 | 0 | 29,62 | 165 | 11,90 |
| PATIENT81 | 1 | 23,95 | 96 | 4,62 |
| PATIENT82 | 0 | 34,15 | 134 | 13,72 |
| PATIENT83 | 1 | 25,22 | 80 | 6,37 |
| PATIENT84 | 0 | 32,65 | 148 | 18,82 |
| PATIENT85 | 1 | 27,93 | 77 | 9,06 |
| PATIENT86 | 1 | 26,29 | 140 | 7,55 |
| PATIENT87 | 0 | 26,37 | 199 | 7,92 |
| PATIENT88 | 0 | 27,76 | 87 | 17,42 |
| PATIENT89 | 0 | 26,26 | 110 | 12,60 |
| PATIENT90 | 0 | 28,08 | 91 | 4,64 |
| PATIENT91 | 1 | 17,72 |  | 8,66 |
| PATIENT92 | 0 | 31,25 | 107 | 10,03 |
| PATIENT93 | 1 | 22,63 | 74 | 2,67 |
| PATIENT94 | 1 | 18,31 | 83 | 3,29 |
| PATIENT95 | 1 | 26,75 | 126 | 9,30 |
| PATIENT96 | 1 | 29,64 | 86 | 12,78 |
| PATIENT97 | 1 | 38,16 | 98 | 9,16 |
| PATIENT98 | 0 | 23,81 | 100 | 12,96 |
| PATIENT99 | 0 | 22,60 | 148 | 18,07 |
| PATIENT100 | 1 | 22,66 | 73 | 5,19 |
| PATIENT101 | 0 | 35,02 | 93 | 8,66 |
| PATIENT102 | 1 | 31,77 | 84 | 4,86 |
| PATIENT103 | 1 | 23,05 | 109 | 3,23 |
| PATIENT104 | 0 | 26,53 | 93 | 6,37 |
| PATIENT105 | 1 | 23,05 | 103 | 4,03 |
| PATIENT106 | 1 | 22,37 | 87 | 6,14 |
| PATIENT107 | 0 | 26,42 | 72 | 10,35 |
| PATIENT108 | 1 | 20,20 | 74 | 6,67 |
| PATIENT109 | 0 | 32,87 | 98 | 3,59 |
| PATIENT110 | 0 | 29,76 | 84 | 7,51 |
| PATIENT111 | 0 | 25,39 | 76 | 7,78 |
| PATIENT112 | 0 | 28,90 | 91 | 7,23 |
| PATIENT113 | 0 | 30,28 | 97 | 13,27 |
| PATIENT114 | 1 | 36,16 | 82 | 7,54 |
| PATIENT115 | 1 | 26,04 | 199 | 11,59 |
| PATIENT116 | 0 | 30,12 | 181 | 19,96 |
| PATIENT117 | 0 | 34,02 | 130 | 16,18 |
| PATIENT118 | 1 | 20,82 | 79 | 5,39 |
| PATIENT119 | 0 | 33,79 | 119 | 13,68 |
| PATIENT120 | 0 | 27,47 | 86 | 5,12 |
| PATIENT121 | 1 | 35,01 | 94 | 5,65 |
| PATIENT122 | 1 | 21,97 | 95 | 4,18 |
| PATIENT123 | 0 | 23,51 | 93 | 19,58 |
| PATIENT124 | 0 | 25,60 | 96 | 6,17 |
| PATIENT125 | 0 | 33,95 | 111 | 7,63 |
| PATIENT126 | 0 | 30,25 | 114 | 9,39 |
| PATIENT127 | 0 | 34,03 | 77 | 8,78 |
| PATIENT128 | 1 | 20,70 | 84 | 4,32 |
| PATIENT129 | 1 | 21,97 | 76 | 1,97 |
| PATIENT130 | 0 | 37,35 | 84 | 7,40 |
| PATIENT131 | 0 | 44,47 | 94 | 10,64 |
| PATIENT132 | 1 | 34,19 | 97 | 5,28 |
| PATIENT133 | 0 | 31,91 | 161 | 12,69 |
| PATIENT134 | 1 | 41,86 | 115 | 9,74 |
| PATIENT135 | 0 | 27,34 | 159 | 5,35 |
| PATIENT136 | 1 | 24,91 | 80 | 5,60 |
| PATIENT137 | 1 | 19,93 | 73 | 8,00 |
| PATIENT138 | 0 | 40,01 | 130 | 12,33 |
| PATIENT139 | 1 | 18,07 | 79 | 6,21 |
| PATIENT140 | 0 | 24,57 |  | 16,71 |
| PATIENT141 | 0 | 28,36 | 87 | 7,29 |
| PATIENT142 | 1 | 29,14 | 84 | 18,29 |
| PATIENT143 | 0 | 33,20 | 128 | 12,75 |
| PATIENT144 | 0 | 33,63 | 164 | 8,16 |
| PATIENT145 | 0 | 30,42 | 155 | 30,95 |
| PATIENT146 | 1 | 28,00 | 94 | 5,26 |
| PATIENT147 | 1 | 39,67 | 96 | 8,38 |
| PATIENT148 | 0 | 28,73 | 119 | 7,87 |
| PATIENT149 | 1 | 25,59 | 240 | 9,88 |
| PATIENT150 | 1 | 27,05 | 83 | 5,20 |
| PATIENT151 | 1 | 24,09 | 81 | 4,46 |
| PATIENT152 | 1 | 21,67 | 84 | 6,76 |
| PATIENT153 | 1 | 24,24 | 83 | 6,70 |
| PATIENT154 | 1 | 47,07 | 84 | 9,38 |
| PATIENT155 | 1 | 23,83 | 79 | 12,82 |
| PATIENT156 | 1 | 31,64 | 78 | 8,99 |
| PATIENT157 | 1 | 37,02 | 131 | 9,32 |
| PATIENT158 | 1 | 14,84 | 85 | 5,73 |
| PATIENT159 | 1 | 26,79 | 111 | 10,92 |
| PATIENT160 | 1 | 29,33 | 90 | 3,29 |
| PATIENT161 | 1 | 27,11 | 92 | 5,27 |
| PATIENT162 | 1 | 34,52 | 114 | 5,42 |
| PATIENT163 | 0 | 30,42 | 98 | 6,91 |
| PATIENT164 | 0 | 28,08 | 87 | 5,10 |
| PATIENT165 | 1 | 40,63 | 197 | 6,82 |
| PATIENT166 | 1 | 27,77 | 101 | 10,40 |
| PATIENT167 | 0 | 25,50 | 101 | 4,94 |
| PATIENT168 | 0 | 26,00 | 81 | 6,97 |
| PATIENT169 | 1 | 17,51 | 80 | 2,84 |
| PATIENT170 | 0 | 24,98 | 136 | 11,56 |
| PATIENT171 | 0 | 28,83 | 140 | 7,89 |
| PATIENT172 | 1 | 23,31 | 84 | 7,18 |
| PATIENT173 | 0 | 32,87 | 111 | 13,49 |
| PATIENT174 | 0 | 26,81 | 92 | 13,41 |
| PATIENT175 | 0 | 29,55 | 97 | 15,67 |
| PATIENT176 | 1 | 22,07 | 102 | 4,56 |
| PATIENT177 | 1 | 29,50 | 99 | 8,61 |
| PATIENT178 | 0 | 22,25 | 79 | 11,85 |
| PATIENT179 | 0 | 29,28 | 262 | 10,72 |
| PATIENT180 | 1 | 21,83 | 88 | 3,05 |
| PATIENT181 | 0 | 36,63 | 85 | 7,17 |
| PATIENT182 | 0 | 25,86 | 87 | 17,25 |
| PATIENT183 | 0 | 31,20 | 91 | 8,17 |
| PATIENT184 | 1 | 44,86 | 81 | 16,58 |
| PATIENT185 | 0 | 27,15 | 86 | 6,32 |
| PATIENT186 | 1 | 23,59 | 115 | 8,17 |
| PATIENT187 | 0 | 26,99 | 80 | 10,39 |
| PATIENT188 | 1 | 20,55 | 177 | 4,47 |
| PATIENT189 | 1 | 36,73 | 92 | 7,03 |
| PATIENT190 | 0 | 32,24 | 91 | 10,35 |
| PATIENT191 | 1 | 24,00 | 103 | 3,97 |
| PATIENT192 | 0 | 24,65 | 89 | 5,02 |
| PATIENT193 | 1 | 29,14 | 96 | 5,85 |
| PATIENT194 | 1 | 20,24 | 95 | 3,17 |
| PATIENT195 | 0 | 15,26 | 69 | 8,41 |
| PATIENT196 | 1 | 23,33 | 89 | 11,21 |
| PATIENT197 | 1 | 35,80 | 104 | 7,64 |
| PATIENT198 | 0 | 31,64 | 217 | 7,82 |
| PATIENT199 | 1 | 33,56 | 81 | 11,13 |
| PATIENT200 | 1 | 25,78 | 142 | 11,66 |
| PATIENT201 | 1 | 21,09 | 77 | 3,33 |
| PATIENT202 | 1 | 30,46 | 121 | 9,23 |
| PATIENT203 | 0 | 26,78 | 112 | 17,03 |
| PATIENT204 | 1 | 28,84 | 90 | 13,13 |
| PATIENT205 | 1 | 26,56 | 156 | 9,99 |
| PATIENT206 | 1 | 16,53 | 69 | 12,81 |
| PATIENT207 | 1 | 33,66 | 82 | 5,62 |
| PATIENT208 | 0 | 26,61 | 105 | 8,24 |
| PATIENT209 | 1 | 28,76 | 84 | 10,39 |
| PATIENT210 | 1 | 32,00 | 104 | 6,72 |
| PATIENT211 | 0 | 27,22 | 122 | 9,47 |
| PATIENT212 | 0 | 31,77 | 210 | 22,28 |
| PATIENT213 | 0 | 32,28 | 99 | 10,46 |
| PATIENT214 | 0 | 26,95 | 120 | 20,99 |
| PATIENT215 | 0 | 35,93 | 87 | 18,80 |
| PATIENT216 | 0 | 28,41 | 101 | 9,94 |
| PATIENT217 | 0 | 25,26 | 169 | 6,74 |
| PATIENT218 | 0 | 30,12 | 116 | 13,30 |
| PATIENT219 | 0 | 30,69 | 89 | 10,98 |
| PATIENT220 | 1 | 33,08 | 96 | 7,73 |
| PATIENT221 | 0 | 35,92 | 99 | 25,74 |
| PATIENT222 | 0 | 30,97 | 88 | 16,38 |
| PATIENT223 | 1 | 32,84 | 105 | 9,97 |
| PATIENT224 | 0 | 18,34 | 81 | 7,65 |
| PATIENT225 | 1 | 21,44 | 81 | 3,70 |
| PATIENT226 | 0 | 30,69 | 102 | 19,33 |
| PATIENT227 | 1 | 27,34 | 98 | 5,69 |
| PATIENT228 | 1 | 42,58 | 125 | 8,54 |
| PATIENT229 | 1 | 23,14 | 120 | 5,84 |
| PATIENT230 | 0 | 21,74 | 91 | 6,86 |
| PATIENT231 | 0 | 35,75 | 128 | 15,13 |
| PATIENT232 | 1 | 39,30 | 99 | 5,93 |
| PATIENT233 | 0 | 32,45 | 120 | 9,31 |
| PATIENT234 | 1 | 23,95 | 72 | 5,83 |
| PATIENT235 | 1 | 23,39 | 79 | 2,64 |
| PATIENT236 | 0 | 29,05 | 145 | 8,58 |
| PATIENT237 | 1 | 23,44 | 104 | 12,78 |
| PATIENT238 | 1 | 24,61 | 75 | 3,42 |
| PATIENT239 | 0 | 33,71 | 89 | 7,83 |
| PATIENT240 | 1 | 25,97 | 92 | 10,71 |
| PATIENT241 | 1 | 27,19 | 82 | 6,92 |
| PATIENT242 | 0 | 36,06 | 143 | 11,31 |
| PATIENT243 | 1 | 34,38 | 179 | 6,46 |
| PATIENT244 | 1 | 20,23 | 84 | 2,19 |
| PATIENT245 | 0 | 29,40 | 91 | 8,21 |
| PATIENT246 | 0 | 28,91 | 93 | 9,30 |
| PATIENT247 | 0 | 25,71 | 90 | 8,79 |
| PATIENT248 | 0 | 33,27 | 140 | 10,10 |
| PATIENT249 | 1 | 31,87 | 128 | 7,58 |
| PATIENT250 | 1 | 21,45 | 229 | 6,57 |
| PATIENT251 | 1 | 37,66 | 83 | 8,41 |
| PATIENT252 | 1 | 26,04 | 79 | 7,16 |
| PATIENT253 | 1 | 23,83 | 95 | 6,64 |
| PATIENT254 | 1 | 38,06 | 151 | 7,56 |
| PATIENT255 | 1 | 22,27 | 86 | 2,88 |
| PATIENT256 | 1 | 21,63 | 80 | 3,25 |
| PATIENT257 | 0 | 31,40 | 104 | 20,20 |
| PATIENT258 | 0 | 30,82 | 134 | 22,36 |
| PATIENT259 | 1 | 42,04 | 143 | 10,71 |
| PATIENT260 | 1 | 26,64 | 84 | 6,40 |
| PATIENT261 | 0 | 33,98 | 100 | 7,48 |
| PATIENT262 | 1 | 29,71 | 74 | 11,78 |
| PATIENT263 | 0 | 26,99 | 90 | 9,97 |
| PATIENT264 | 0 | 27,13 | 130 | 8,09 |
| PATIENT265 | 1 | 22,67 | 120 | 6,54 |
| PATIENT266 | 0 | 32,00 | 96 | 12,47 |
| PATIENT267 | 0 | 34,80 | 215 | 7,07 |
| PATIENT268 | 0 | 43,49 | 92 | 7,30 |
| PATIENT269 | 0 | 18,45 | 191 | 8,75 |
| PATIENT270 | 0 | 29,67 | 86 | 5,75 |
| PATIENT271 | 0 | 22,20 | 80 | 9,88 |
| PATIENT272 | 1 | 25,54 | 123 | 5,81 |
| PATIENT273 | 0 | 26,99 | 82 | 9,45 |
| PATIENT274 | 1 | 25,81 | 93 | 7,19 |
| PATIENT275 | 1 | 25,91 | 93 | 4,10 |
| PATIENT276 | 0 | 24,49 | 145 | 10,30 |
| PATIENT277 | 1 | 35,79 | 101 | 6,64 |
| PATIENT278 | 0 | 36,33 | 92 | 15,33 |
| PATIENT279 | 1 | 27,00 | 92 | 3,08 |
| PATIENT280 | 0 | 22,39 | 82 | 4,95 |
| PATIENT281 | 1 | 27,14 | 86 | 8,03 |
| PATIENT282 | 1 | 31,59 | 97 | 14,03 |
| PATIENT283 | 1 | 17,99 | 75 | 8,60 |
| PATIENT284 | 1 | 26,37 | 82 | 6,84 |
| PATIENT285 | 0 | 26,95 | 139 | 6,71 |
| PATIENT286 | 0 | 22,20 | 80 | 9,88 |
| PATIENT287 | 0 | 23,88 | 93 | 9,91 |
| PATIENT288 | 1 | 27,70 | 99 | 4,30 |
| PATIENT289 | 1 | 22,27 | 83 | 3,87 |
| PATIENT290 | 0 | 27,97 | 114 | 17,69 |
| PATIENT291 | 1 | 33,56 | 95 | 6,19 |
| PATIENT292 | 0 | 28,65 | 117 | 4,97 |
| PATIENT293 | 0 | 24,80 | 131 | 6,23 |
| PATIENT294 | 1 | 23,38 | 83 | 5,44 |
| PATIENT295 | 1 | 32,74 | 80 | 8,42 |
| PATIENT296 | 0 | 26,85 | 94 | 4,39 |
| PATIENT297 | 1 | 39,06 | 93 | 7,98 |
| PATIENT298 | 0 | 22,57 | 77 | 7,28 |
| PATIENT299 | 0 | 34,03 | 302 | 5,65 |
| PATIENT300 | 0 | 32,41 | 147 | 11,08 |
| PATIENT301 | 1 | 23,20 | 77 | 3,88 |
| PATIENT302 | 0 | 24,96 | 88 | 9,96 |
| PATIENT303 | 1 | 26,37 | 91 | 2,70 |
| PATIENT304 | 1 | 29,38 | 96 | 9,44 |
| PATIENT305 | 0 | 37,04 | 80 | 12,32 |
| PATIENT306 | 1 | 19,23 | 82 | 7,92 |
| PATIENT307 | 1 | 30,32 | 82 | 3,65 |
| PATIENT308 | 1 | 16,82 | 80 | 5,13 |
| PATIENT309 | 1 | 33,66 | 94 | 9,74 |
| PATIENT310 | 0 | 24,24 | 95 | 6,05 |
| PATIENT311 | 0 | 23,38 | 85 | 4,36 |
| PATIENT312 | 0 | 26,67 | 222 | 26,52 |
| PATIENT313 | 1 | 38,20 | 299 | 6,40 |
| PATIENT314 | 1 | 22,27 | 88 | 4,82 |
| PATIENT315 | 0 | 28,52 | 118 | 10,53 |
| PATIENT316 | 1 | 30,22 | 197 | 2,54 |
| PATIENT317 | 1 | 37,34 | 122 | 6,87 |
| PATIENT318 | 1 | 29,58 | 78 | 15,84 |
| PATIENT319 | 1 | 31,98 | 118 | 3,82 |
| PATIENT320 | 0 | 37,29 | 97 | 9,06 |
| PATIENT321 | 1 | 36,33 | 83 | 6,02 |
| PATIENT322 | 1 | 20,00 | 121 | 4,28 |
| PATIENT323 | 0 | 24,91 | 96 | 10,58 |
| PATIENT324 | 0 | 28,71 | 108 | 9,06 |
| PATIENT325 | 0 | 22,39 | 90 | 8,43 |
| PATIENT326 | 0 | 30,72 | 107 | 5,91 |
| PATIENT327 | 0 | 27,68 | 87 | 11,16 |
| PATIENT328 | 0 | 21,13 | 83 | 5,85 |
| PATIENT329 | 1 | 33,59 | 54 | 6,12 |
| PATIENT330 | 0 | 27,68 | 105 | 5,68 |
| PATIENT331 | 0 | 29,73 | 164 | 7,77 |
| PATIENT332 | 0 | 30,35 | 88 | 5,59 |
| PATIENT333 | 0 | 29,07 | 98 | 11,50 |
| PATIENT334 | 0 | 23,08 | 74 | 6,11 |
| PATIENT335 | 0 | 18,78 | 79 | 7,48 |
| PATIENT336 | 0 | 32,70 | 134 | 4,18 |
| PATIENT337 | 1 | 25,13 | 116 | 4,50 |
| PATIENT338 | 0 | 24,20 | 90 | 8,93 |
| PATIENT339 | 0 | 32,70 | 134 | 5,92 |
| PATIENT340 | 1 | 21,48 | 76 | 5,22 |
| PATIENT341 | 1 | 23,44 | 92 | 4,31 |
| PATIENT342 | 1 | 33,33 | 318 | 5,98 |
| PATIENT343 | 0 | 27,28 | 141 | 15,95 |
| PATIENT344 | 1 | 17,97 | 84 | 3,37 |
| PATIENT345 | 1 | 30,22 | 84 | 9,88 |
| PATIENT346 | 1 | 37,66 | 97 | 10,16 |
| PATIENT347 | 0 | 51,12 | 70 | 7,25 |
| PATIENT348 | 1 | 36,42 | 102 | 6,72 |
| PATIENT349 | 1 | 16,90 | 76 | 7,00 |
| PATIENT350 | 0 | 21,31 | 76 | 6,57 |
| PATIENT351 | 1 | 21,77 | 77 | 3,87 |
| PATIENT352 | 1 | 23,54 | 84 | 3,98 |
| PATIENT353 | 0 | 30,67 | 103 | 7,35 |
| PATIENT354 | 0 | 26,86 | 96 | 7,86 |
| PATIENT355 | 1 | 20,59 | 80 | 3,31 |
| PATIENT356 | 0 | 26,99 | 67 | 22,72 |
| PATIENT357 | 0 | 35,57 | 297 | 18,90 |
| PATIENT358 | 0 | 36,00 | 159 | 16,64 |
| PATIENT359 | 0 | 27,68 | 87 | 6,85 |
| PATIENT360 | 0 | 35,82 | 285 | 14,98 |
| PATIENT361 | 0 | 23,53 | 104 | 9,17 |
| PATIENT362 | 0 | 17,24 | 94 | 2,94 |
| PATIENT363 | 1 | 23,73 | 86 | 7,03 |
| PATIENT364 | 0 | 21,71 | 127 | 9,42 |
| PATIENT365 | 1 | 34,69 | 131 | 7,85 |
| PATIENT366 | 1 | 19,96 | 86 | 7,26 |
| PATIENT367 | 0 | 24,03 | 125 | 8,64 |
| PATIENT368 | 1 | 19,40 | 79 | 4,93 |
| PATIENT369 | 0 | 22,41 | 74 | 15,98 |
| PATIENT370 | 1 | 23,73 | 94 | 6,37 |
| PATIENT371 | 0 | 32,21 | 134 | 14,69 |
| PATIENT372 | 1 | 40,40 | 108 | 12,80 |
| PATIENT373 | 0 | 26,23 | 118 | 9,88 |
| PATIENT374 | 0 | 26,99 | 106 | 12,47 |
| PATIENT375 | 0 | 22,82 | 112 | 14,82 |
| PATIENT376 | 1 | 18,52 | 121 | 4,87 |
| PATIENT377 | 1 | 27,92 | 74 | 8,78 |
| PATIENT378 | 0 | 32,41 | 98 | 11,58 |
| PATIENT379 | 1 | 24,65 | 94 | 3,26 |
| PATIENT380 | 0 | 31,53 | 187 | 8,75 |
| PATIENT381 | 1 | 18,67 | 71 | 2,69 |
| PATIENT382 | 1 | 18,65 | 80 | 4,96 |
| PATIENT383 | 1 | 26,08 | 79 | 7,98 |
| PATIENT384 | 0 | 31,83 | 95 | 9,76 |
| PATIENT385 | 0 | 24,62 | 113 | 5,61 |
| PATIENT386 | 0 | 26,12 | 93 | 7,37 |
| PATIENT387 | 1 | 26,99 | 113 | 4,89 |
| PATIENT388 | 1 | 18,97 | 85 | 10,30 |
| PATIENT389 | 1 | 23,60 | 73 | 4,02 |
| PATIENT390 | 1 | 22,77 | 73 | 5,03 |
| PATIENT391 | 1 | 31,64 | 111 | 6,90 |
| PATIENT392 | 1 | 24,44 | 105 | 3,62 |
| PATIENT393 | 1 | 23,31 | 79 | 4,05 |
| PATIENT394 | 1 | 24,56 | 76 | 9,13 |
| PATIENT395 | 0 | 27,87 | 136 | 8,89 |
| PATIENT396 | 0 | 29,04 | 139 | 11,26 |
| PATIENT397 | 0 | 31,25 | 112 | 12,38 |
| PATIENT398 | 1 | 23,05 | 110 | 5,64 |
| PATIENT399 | 1 | 40,00 | 123 | 7,06 |
| PATIENT400 | 1 | 23,05 | 88 | 2,35 |
| PATIENT401 | 0 | 34,31 | 96 | 5,93 |
| PATIENT402 | 0 | 29,59 | 78 | 6,09 |
| PATIENT403 | 0 | 27,68 | 109 | 14,19 |
| PATIENT404 | 1 | 34,89 | 122 | 6,46 |
| PATIENT405 | 0 | 23,55 | 117 | 2,75 |
| PATIENT406 | 1 | 36,98 | 320 | 12,08 |
| PATIENT407 | 1 | 24,34 | 84 | 5,35 |
| PATIENT408 | 1 | 28,15 | 310 | 7,21 |
| PATIENT409 | 1 | 24,39 | 80 | 8,20 |
| PATIENT410 | 1 | 31,60 | 91 | 10,08 |
| PATIENT411 | 0 | 25,85 | 84 | 9,19 |
| PATIENT412 | 1 | 22,15 | 84 | 3,41 |
| PATIENT413 | 1 | 28,30 | 82 | 25,45 |
| PATIENT414 | 0 | 32,65 | 88 | 11,95 |
| PATIENT415 | 1 | 28,71 | 84 | 5,72 |
| PATIENT416 | 1 | 20,44 | 119 | 6,25 |
| PATIENT417 | 0 | 32,98 | 114 | 17,61 |
| PATIENT418 | 0 | 29,94 | 90 | 11,53 |
| PATIENT419 | 0 | 48,93 | 121 | 7,40 |
| PATIENT420 | 0 | 28,72 | 111 | 17,83 |
| PATIENT421 | 1 | 19,84 | 81 | 4,42 |
| PATIENT422 | 0 | 23,10 | 80 | 15,25 |
| PATIENT423 | 1 | 35,16 | 122 | 12,51 |
| PATIENT424 | 1 | 23,31 | 76 | 6,97 |
| PATIENT425 | 1 | 37,02 | 73 | 10,84 |
| PATIENT426 | 0 | 30,40 | 96 | 10,01 |
| PATIENT427 | 0 | 22,09 | 105 | 4,54 |
| PATIENT428 | 1 | 24,91 | 72 | 9,64 |
| PATIENT429 | 0 | 26,05 | 91 | 4,03 |
| PATIENT430 | 0 | 32,95 | 102 | 9,39 |
| PATIENT431 | 1 | 43,06 | 129 | 19,26 |
| PATIENT432 | 0 | 28,37 | 82 | 10,25 |
| PATIENT433 | 1 | 26,08 | 129 | 13,19 |
| PATIENT434 | 0 | 25,00 | 163 | 7,59 |
| PATIENT435 | 0 | 28,90 | 88 | 7,61 |
| PATIENT436 | 1 | 22,43 | 77 | 3,67 |
| PATIENT437 | 1 | 34,42 | 157 | 11,95 |
| PATIENT438 | 1 | 22,89 | 81 | 4,57 |
| PATIENT439 | 0 | 20,99 | 78 | 5,41 |
| PATIENT440 | 1 | 33,40 | 133 | 9,67 |
| PATIENT441 | 0 | 37,32 | 101 | 14,00 |
| PATIENT442 | 0 | 25,61 | 101 | 15,84 |
| PATIENT443 | 1 | 21,01 | 99 | 5,91 |
| PATIENT444 | 0 | 45,63 | 133 | 6,84 |
| PATIENT445 | 0 | 34,84 | 117 | 3,79 |
| PATIENT446 | 0 | 24,93 | 85 | 5,96 |
| PATIENT447 | 1 | 29,13 | 83 | 5,09 |
| PATIENT448 | 0 | 25,10 | 100 | 3,73 |
| PATIENT449 | 0 | 23,43 | 118 | 6,56 |
| PATIENT450 | 0 | 25,73 | 118 | 8,70 |
| PATIENT451 | 1 | 22,96 | 102 | 5,21 |
| PATIENT452 | 0 | 26,35 | 74 | 14,10 |
| PATIENT453 | 0 | 33,30 | 95 | 10,00 |
| PATIENT454 | 0 | 31,38 | 91 | 8,61 |
| PATIENT455 | 0 | 33,33 | 94 | 7,54 |
| PATIENT456 | 0 | 22,50 | 91 | 12,25 |
| PATIENT457 | 1 | 18,29 | 76 | 7,20 |
| PATIENT458 | 1 | 27,24 | 87 | 4,17 |
| PATIENT459 | 0 | 31,18 | 109 | 8,12 |
| PATIENT460 | 0 | 20,75 | 91 | 4,91 |
| PATIENT461 | 1 | 22,15 | 91 | 4,96 |
| PATIENT462 | 0 | 26,15 | 86 | 7,15 |
| PATIENT463 | 1 | 21,33 | 86 | 4,66 |
| PATIENT464 | 1 | 33,53 | 102 | 10,10 |
| PATIENT465 | 0 | 28,32 | 112 | 13,25 |
| PATIENT466 | 1 | 26,04 | 146 | 17,97 |
| PATIENT467 | 0 | 30,48 | 91 | 9,79 |
| PATIENT468 | 1 | 34,76 | 112 | 7,24 |
| PATIENT469 | 1 | 35,09 | 125 | 8,85 |
| PATIENT470 | 0 | 22,49 | 88 | 5,54 |
| PATIENT471 | 1 | 25,48 | 90 | 8,52 |
| PATIENT472 | 1 | 20,57 | 79 | 5,68 |
| PATIENT473 | 0 | 29,67 | 120 | 12,25 |
| PATIENT474 | 1 | 23,05 | 81 | 6,06 |
| PATIENT475 | 0 | 24,34 | 100 | 6,44 |
| PATIENT476 | 1 | 23,88 | 133 | 5,93 |
| PATIENT477 | 0 | 27,10 | 94 | 12,23 |
| PATIENT478 | 0 | 25,24 | 99 | 4,14 |
| PATIENT479 | 0 | 23,44 | 114 | 7,29 |
| PATIENT480 | 1 | 42,86 | 99 | 7,60 |
| PATIENT481 | 0 | 27,82 | 97 | 4,72 |
| PATIENT482 | 0 | 29,09 | 100 | 8,01 |
| PATIENT483 | 1 | 21,12 | 77 | 7,07 |
| PATIENT484 | 1 | 32,62 | 95 | 12,21 |
| PATIENT485 | 1 | 25,96 | 127 | 6,11 |
| PATIENT486 | 1 | 27,54 | 90 | 5,75 |
| PATIENT487 | 1 | 32,03 | 86 | 2,97 |
| PATIENT488 | 1 | 31,84 | 90 | 4,63 |
| PATIENT489 | 0 | 24,22 | 78 | 11,46 |
| PATIENT490 | 0 | 24,49 | 93 | 16,03 |
| PATIENT491 | 1 | 19,14 | 80 | 5,10 |
| PATIENT492 | 0 | 32,41 | 108 | 3,14 |
| PATIENT493 | 0 | 22,69 | 84 | 6,10 |
| PATIENT494 | 0 | 23,56 | 86 | 3,70 |
| PATIENT495 | 0 | 26,54 | 88 | 6,35 |
| PATIENT496 | 0 | 28,06 | 99 | 7,51 |
| PATIENT497 | 1 | 20,06 | 74 | 6,23 |
| PATIENT498 | 1 | 30,26 | 99 | 4,73 |
| PATIENT499 | 1 | 30,67 | 142 | 15,95 |
| PATIENT500 | 1 | 18,73 | 72 | 3,62 |
| PATIENT501 | 1 | 23,05 | 86 | 4,33 |
| PATIENT502 | 1 | 19,26 | 77 | 3,99 |
| PATIENT503 | 1 | 42,44 |  | 13,79 |
| PATIENT504 | 1 | 27,06 | 95 | 4,70 |
| PATIENT505 | 1 | 28,54 | 118 | 9,25 |
| PATIENT506 | 1 | 28,41 | 102 | 7,79 |
| PATIENT507 | 0 | 29,76 | 99 | 7,59 |
| PATIENT508 | 1 | 26,49 | 242 | 3,25 |
| PATIENT509 | 1 | 21,80 | 85 | 7,07 |
| PATIENT510 | 1 | 33,33 | 119 | 9,66 |
| PATIENT511 | 1 | 27,29 | 92 | 5,90 |
| PATIENT512 | 1 | 31,22 | 79 | 11,89 |
| PATIENT513 | 1 | 20,76 | 80 | 3,10 |
| PATIENT514 | 0 | 29,04 | 104 | 11,05 |
| PATIENT515 | 1 | 25,48 | 144 | 4,32 |
| PATIENT516 | 0 | 24,45 | 134 | 9,86 |
| PATIENT517 | 1 | 32,01 | 100 | 8,30 |
| PATIENT518 | 1 | 25,39 | 83 | 2,95 |
| PATIENT519 | 1 | 39,73 | 68 | 11,31 |
| PATIENT520 | 1 | 21,80 | 78 | 4,15 |
| PATIENT521 | 0 | 19,27 | 80 | 8,91 |
| PATIENT522 | 1 | 25,12 | 80 | 3,97 |
| PATIENT523 | 1 | 26,54 | 325 | 20,61 |
| PATIENT524 | 0 | 24,80 | 140 | 5,68 |
| PATIENT525 | 1 | 19,88 | 81 | 3,51 |
| PATIENT526 | 1 | 25,97 | 97 | 3,05 |
| PATIENT527 | 0 | 22,29 | 85 | 8,15 |
| PATIENT528 | 0 | 27,10 | 106 | 14,54 |
| PATIENT529 | 0 | 24,69 | 81 | 8,31 |
| PATIENT530 | 0 | 27,06 | 71 | 6,04 |
| PATIENT531 | 0 | 32,24 | 94 | 9,63 |
| PATIENT532 | 0 | 21,51 | 78 | 4,40 |
| PATIENT533 | 1 | 18,93 | 77 | 5,26 |
| PATIENT534 | 1 | 32,69 | 95 | 9,60 |
| PATIENT535 | 1 | 23,63 | 137 | 4,41 |
| PATIENT536 | 1 | 17,29 | 82 | 4,54 |
| PATIENT537 | 0 | 26,81 | 92 | 6,01 |
| PATIENT538 | 0 | 35,08 | 108 | 6,87 |
| PATIENT539 | 0 | 26,72 | 72 | 9,28 |
| PATIENT540 | 0 | 24,01 | 115 | 8,69 |
| PATIENT541 | 1 | 40,16 | 131 | 5,23 |
| PATIENT542 | 1 | 37,34 | 138 | 9,68 |
| PATIENT543 | 1 | 18,36 | 86 | 4,18 |
| PATIENT544 | 0 | 27,48 | 134 | 9,98 |
| PATIENT545 | 0 | 25,97 | 164 | 11,27 |
| PATIENT546 | 1 | 27,70 | 78 | 4,70 |
| PATIENT547 | 1 | 32,05 | 107 | 7,90 |
| PATIENT548 | 0 | 25,56 | 55 | 9,86 |
| PATIENT549 | 0 | 27,55 | 129 | 7,63 |
| PATIENT550 | 1 | 21,21 | 78 | 4,31 |
| PATIENT551 | 1 | 21,05 | 82 | 5,61 |
| PATIENT552 | 1 | 27,10 | 107 | 5,83 |
| PATIENT553 | 0 | 30,00 | 81 | 19,08 |
| PATIENT554 | 0 | 26,56 | 87 | 11,63 |
| PATIENT555 | 1 | 34,02 | 89 | 4,66 |
| PATIENT556 | 0 | 24,03 | 85 | 4,96 |
| PATIENT557 | 0 | 28,86 | 90 | 22,20 |
| PATIENT558 | 0 | 27,77 | 165 | 15,48 |
| PATIENT559 | 0 | 24,16 | 138 | 8,69 |
| PATIENT560 | 0 | 26,78 | 73 | 8,50 |
| PATIENT561 | 0 | 24,84 | 81 | 4,85 |
| PATIENT562 | 1 | 25,13 | 175 | 9,18 |
| PATIENT563 | 0 | 22,04 | 109 | 21,89 |
| PATIENT564 | 0 | 26,89 | 98 | 9,47 |
| PATIENT565 | 0 | 27,78 | 87 | 9,44 |
| PATIENT566 | 0 | 29,41 | 100 | 7,98 |
| PATIENT567 | 0 | 40,04 | 89 | 10,50 |
| PATIENT568 | 0 | 40,04 | 89 | 10,50 |
| PATIENT569 | 1 | 35,15 | 126 | 8,81 |
| PATIENT570 | 1 | 19,36 | 78 | 4,94 |
| PATIENT571 | 0 | 29,22 | 83 | 6,52 |
| PATIENT572 | 0 | 27,41 | 106 | 6,66 |
| PATIENT573 | 0 | 21,46 | 94 | 3,34 |
| PATIENT574 | 1 | 31,15 | 97 | 6,83 |
| PATIENT575 | 0 | 29,40 | 105 | 4,64 |
| PATIENT576 | 0 | 30,12 | 109 | 5,87 |
| PATIENT577 | 1 | 24,89 | 80 | 10,44 |
| PATIENT578 | 0 | 24,77 | 83 | 8,82 |
| PATIENT579 | 1 | 27,97 | 91 | 8,40 |
| PATIENT580 | 1 | 19,49 | 73 | 5,62 |
| PATIENT581 | 0 | 28,09 | 90 | 7,56 |
| PATIENT582 | 0 | 30,45 | 89 | 13,55 |
| PATIENT583 | 1 | 30,48 | 103 | 5,47 |
| PATIENT584 | 0 | 26,30 | 102 | 4,57 |
| PATIENT585 | 0 | 32,21 | 94 | 12,21 |
| PATIENT586 | 0 | 26,99 | 94 | 5,06 |
| PATIENT587 | 0 | 29,40 | 122 | 12,45 |
| PATIENT588 | 0 | 25,42 | 82 | 9,85 |
| PATIENT589 | 0 | 33,33 | 104 | 10,28 |
| PATIENT590 | 1 | 27,39 | 87 | 4,30 |
| PATIENT591 | 0 | 24,22 | 92 | 5,98 |
| PATIENT592 | 0 | 27,17 | 85 | 9,27 |
| PATIENT593 | 0 | 27,76 | 153 | 9,91 |
| PATIENT594 | 0 | 24,22 | 156 | 6,05 |
| PATIENT595 | 0 | 28,08 | 134 | 9,89 |
| PATIENT596 | 0 | 22,53 | 104 | 8,04 |
| PATIENT597 | 0 | 36,05 | 85 | 11,63 |
| PATIENT598 | 1 | 35,80 | 221 | 11,14 |
| PATIENT599 | 0 | 23,51 | 102 | 11,67 |
| PATIENT600 | 0 | 36,93 | 132 | 12,53 |
| PATIENT601 | 0 | 35,83 | 76 | 10,53 |
| PATIENT602 | 1 | 26,22 | 85 | 5,26 |
| PATIENT603 | 1 | 25,97 | 92 | 5,12 |
| PATIENT604 | 1 | 25,10 | 79 | 2,81 |
| PATIENT605 | 1 | 26,81 | 114 | 7,29 |
| PATIENT606 | 0 | 22,46 | 90 | 4,29 |
| PATIENT607 | 0 | 23,96 | 90 | 4,83 |
| PATIENT608 | 0 | 24,26 | 204 | 6,36 |
| PATIENT609 | 0 | 25,91 | 103 | 9,77 |
| PATIENT610 | 1 | 27,68 | 85 | 1,69 |
| PATIENT611 | 1 | 55,36 | 93 | 12,54 |
| PATIENT612 | 0 | 24,38 | 98 | 5,81 |
| PATIENT613 | 1 | 25,48 | 89 | 6,21 |
| PATIENT614 | 1 | 24,17 | 86 | 3,49 |
| PATIENT615 | 1 | 25,90 | 76 | 4,91 |
| PATIENT616 | 1 | 23,81 | 79 | 4,35 |
| PATIENT617 | 1 | 23,03 | 82 | 9,37 |
| PATIENT618 | 0 | 30,30 | 104 | 9,51 |
| PATIENT619 | 1 | 25,54 | 85 | 5,69 |
| PATIENT620 | 1 | 19,65 | 81 | 12,78 |
| PATIENT621 | 1 | 33,51 | 108 | 7,84 |
| PATIENT622 | 0 | 26,11 | 135 | 25,10 |
| PATIENT623 | 0 | 30,20 | 94 | 13,62 |
| PATIENT624 | 0 | 27,92 | 116 | 6,24 |
| PATIENT625 | 0 | 50,14 | 97 | 15,08 |
| PATIENT626 | 1 | 22,15 | 99 | 9,53 |
| PATIENT627 | 0 | 39,67 | 117 | 2,52 |
| PATIENT628 | 1 | 25,51 | 81 | 3,32 |
| PATIENT629 | 0 | 23,83 | 75 | 11,89 |
| PATIENT630 | 1 | 30,80 | 92 | 7,09 |
| PATIENT631 | 0 | 22,40 | 75 | 11,31 |
| PATIENT632 | 0 | 32,51 | 95 | 9,92 |
| PATIENT633 | 1 | 28,52 | 93 | 12,50 |
| PATIENT634 | 0 | 34,94 | 83 | 17,06 |
| PATIENT635 | 1 | 20,96 | 76 | 3,89 |
| PATIENT636 | 1 | 34,38 | 107 | 15,06 |
| PATIENT637 | 0 | 29,30 | 98 | 6,24 |
| PATIENT638 | 1 | 36,20 | 74 | 12,64 |
| PATIENT639 | 1 | 27,89 | 127 | 5,91 |
| PATIENT640 | 1 | 23,05 | 88 | 6,85 |
| PATIENT641 | 1 | 21,97 | 90 | 4,05 |
| PATIENT642 | 1 | 23,31 | 88 | 3,54 |
| PATIENT643 | 0 | 26,17 | 91 | 2,95 |
| PATIENT644 | 1 | 27,56 | 111 | 12,43 |
| PATIENT645 | 0 | 23,84 | 100,05 | 9,53 |
| PATIENT646 | 0 | 27,41 | 98 | 10,42 |
| PATIENT647 | 1 | 33,76 | 114 | 4,68 |
| PATIENT648 | 1 | 19,23 | 80 | 5,19 |
| PATIENT649 | 0 | 30,48 | 130 | 9,60 |
| PATIENT650 | 1 | 28,70 | 76 | 6,87 |
| PATIENT651 | 1 | 22,50 | 85 | 1,87 |
| PATIENT652 | 0 | 24,68 | 113 | 14,41 |
| PATIENT653 | 1 | 20,57 | 79 | 6,23 |
| PATIENT654 | 0 | 32,93 | 95 | 15,96 |
| PATIENT655 | 1 | 30,67 | 79 | 5,71 |
| PATIENT656 | 0 | 26,64 | 113 | 11,40 |
| PATIENT657 | 1 | 22,68 | 89 | 3,38 |
| PATIENT658 | 0 | 24,91 | 114 | 13,54 |
| PATIENT659 | 1 | 20,20 | 92 | 7,86 |
| PATIENT660 | 0 | 41,18 | 108 | 15,99 |
| PATIENT661 | 1 | 33,76 | 180 | 12,66 |
| PATIENT662 | 0 | 25,20 | 79 | 6,88 |
| PATIENT663 | 1 | 23,08 | 98 | 5,17 |
| PATIENT664 | 0 | 27,17 | 98 | 13,27 |
| PATIENT665 | 0 | 33,41 | 108 | 6,47 |
| PATIENT666 | 1 | 23,31 | 82 | 5,35 |
| PATIENT667 | 0 | 27,14 | 72 | 8,79 |
| PATIENT668 | 0 | 30,45 | 80 | 10,38 |
| PATIENT669 | 1 | 27,34 | 97 | 10,10 |
| PATIENT670 | 1 | 31,45 | 93 | 7,06 |
| PATIENT671 | 0 | 29,54 | 122 | 14,73 |
| PATIENT672 | 1 | 26,81 | 97 | 10,51 |
| PATIENT673 | 1 | 28,67 | 108 | 7,13 |
| PATIENT674 | 1 | 25,63 | 85 | 2,85 |
| PATIENT675 | 0 | 31,17 | 89 | 10,73 |
| PATIENT676 | 1 | 31,38 | 99 | 9,37 |
| PATIENT677 | 1 | 26,44 | 169 | 8,82 |
| PATIENT678 | 1 | 25,91 | 114 | 4,02 |
| PATIENT679 | 0 | 40,92 | 258 | 24,27 |
| PATIENT680 | 1 | 21,37 | 85 | 1,92 |
| PATIENT681 | 0 | 33,06 | 101 | 10,92 |
| PATIENT682 | 0 | 27,68 | 110 | 15,13 |
| PATIENT683 | 0 | 33,90 | 101 | 10,78 |
| PATIENT684 | 0 | 27,07 | 93 | 8,04 |
| PATIENT685 | 1 | 35,01 | 111 | 10,95 |
| PATIENT686 | 0 | 26,85 | 66 | 22,17 |
| PATIENT687 | 0 | 27,44 | 83 | 5,23 |
| PATIENT688 | 1 | 25,82 | 87 | 5,12 |
| PATIENT689 | 0 | 30,37 | 96 | 8,09 |
| PATIENT690 | 1 | 38,10 | 78 | 2,57 |
| PATIENT691 | 0 | 27,64 | 88 | 7,11 |
| PATIENT692 | 1 | 33,75 | 232 | 12,96 |
| PATIENT693 | 0 | 22,84 | 89 | 12,05 |
| PATIENT694 | 0 | 28,65 | 106 | 12,64 |
| PATIENT695 | 1 | 21,88 | 87 | 4,32 |
| PATIENT696 | 0 | 30,27 | 96 | 8,22 |
| PATIENT697 | 1 | 48,89 | 131 | 10,19 |
| PATIENT698 | 1 | 19,47 | 85 | 4,02 |
| PATIENT699 | 1 | 28,51 | 90 | 7,85 |
| PATIENT700 | 0 | 24,92 | 152 | 8,31 |
| PATIENT701 | 1 | 20,05 | 80 | 4,13 |
| PATIENT702 | 0 | 28,74 | 139 | 11,42 |
| PATIENT703 | 1 | 27,34 | 78 | 6,52 |
| PATIENT704 | 0 | 28,57 | 76 | 13,81 |
| PATIENT705 | 0 | 32,10 | 142 | 12,43 |
| PATIENT706 | 0 | 29,32 | 89 | 9,04 |
| PATIENT707 | 0 | 35,27 | 86 | 5,16 |
| PATIENT708 | 0 | 26,37 | 143 | 8,98 |
| PATIENT709 | 0 | 28,83 | 87 | 14,04 |
| PATIENT710 | 0 | 23,51 | 90 | 6,23 |
| PATIENT711 | 0 | 20,52 | 120 | 9,66 |
| PATIENT712 | 1 | 20,90 | 81 | 3,76 |
| PATIENT713 | 1 | 18,50 | 80 | 3,89 |
| PATIENT714 | 0 | 28,01 | 112 | 8,02 |
| PATIENT715 | 0 | 25,14 | 83 | 6,27 |
| PATIENT716 | 0 | 33,82 | 87 | 14,52 |
| PATIENT717 | 0 | 32,60 | 115 | 4,69 |
| PATIENT718 | 0 | 29,36 | 104 | 6,00 |
| PATIENT719 | 0 | 29,06 | 130 | 8,05 |
| PATIENT720 | 0 | 31,46 | 86 | 11,88 |
| PATIENT721 | 1 | 29,76 | 79 | 6,18 |
| PATIENT722 | 0 | 22,76 | 97 | 9,20 |
| PATIENT723 | 1 | 30,44 | 99 | 6,01 |
| PATIENT724 | 0 | 29,06 | 83 | 9,64 |
| PATIENT725 | 0 | 24,91 | 108 | 7,08 |
| PATIENT726 | 0 | 19,59 | 141 | 6,07 |
| PATIENT727 | 0 | 34,29 | 91 | 17,54 |
| PATIENT728 | 0 | 34,72 | 97 | 12,82 |
| PATIENT729 | 0 | 27,46 | 87 | 18,01 |
| PATIENT730 | 0 | 31,84 | 92 | 23,88 |
| PATIENT731 | 0 | 22,02 | 99 | 19,60 |
| PATIENT732 | 0 | 24,42 | 79 | 5,19 |
| PATIENT733 | 0 | 18,90 | 71 | 10,92 |
| PATIENT734 | 0 | 31,35 | 93 | 10,45 |
| PATIENT735 | 0 | 34,40 | 106 | 9,18 |
| PATIENT736 | 0 | 23,12 | 83 | 11,28 |
| PATIENT737 | 0 | 32,87 | 140 | 20,77 |
| PATIENT738 | 1 | 31,58 | 91 | 4,39 |
| PATIENT739 | 1 | 30,26 | 86 | 6,20 |
| PATIENT740 | 0 | 24,11 | 119 | 8,65 |
| PATIENT741 | 0 | 27,04 | 88 | 6,90 |
| PATIENT742 | 0 | 43,43 | 96 | 18,57 |
| PATIENT743 | 1 | 43,26 | 114 | 11,14 |
| PATIENT744 | 1 | 28,72 | 63 | 5,57 |
| PATIENT745 | 0 | 34,06 | 95 | 15,84 |
| PATIENT746 | 1 | 17,65 | 81 | 6,02 |
| PATIENT747 | 1 | 27,47 | 82 | 7,70 |
| PATIENT748 | 1 | 19,53 | 81 | 5,03 |
| PATIENT749 | 0 | 32,11 | 113 | 9,76 |
| PATIENT750 | 1 | 25,90 | 184 | 6,59 |
| PATIENT751 | 0 | 29,72 | 82 | 3,97 |
| PATIENT752 | 0 | 31,91 | 146 | 5,63 |
| PATIENT753 | 0 | 26,88 | 93 | 12,13 |
| PATIENT754 | 0 | 20,18 | 86 | 5,38 |
| PATIENT755 | 0 | 24,49 | 91 | 6,31 |
| PATIENT756 | 1 | 23,15 | 85 | 4,46 |
| PATIENT757 | 0 | 26,49 | 104 | 6,39 |
| PATIENT758 | 1 | 18,07 | 74 | 3,81 |
| PATIENT759 | 1 | 29,07 | 76 | 3,49 |
| PATIENT760 | 0 | 26,12 | 85 | 18,62 |
| PATIENT761 | 1 | 33,33 | 117 | 5,94 |
| PATIENT762 | 1 | 25,26 | 95 | 8,11 |
| PATIENT763 | 1 | 27,34 | 84 | 4,85 |
| PATIENT764 | 1 | 27,10 | 73 | 6,55 |
| PATIENT765 | 1 | 34,63 | 89 | 5,61 |
| PATIENT766 | 1 | 23,10 | 95 | 3,47 |
| PATIENT767 | 1 | 19,57 | 79 | 7,81 |
| PATIENT768 | 0 | 26,45 | 232 | 6,41 |
| PATIENT769 | 1 | 29,07 | 142 | 5,66 |
| PATIENT770 | 1 | 42,69 | 224 | 5,77 |
| PATIENT771 | 0 | 40,74 | 126 | 10,68 |
