# Supplementary material for: Gender, BMI and fasting hyperglycaemia influence Monocyte to-HDL ratio (MHR) index in metabolic subjects
Source: PLoS One. 2020 Apr 28;15(4):e0231927. doi: 10.1371/journal.pone.0231927 (PMC7188261; doi:10.1371/journal.pone.0231927)
Supplement: S2 Table — Values are expressed as mean ±Standard Deviation or median ± [IQR] respectively for normal (*) and non-normal (**) distributed numeric variables, and with n (%) for categorical ones. Abbreviations: Number Obs., number of available data; St.Dev, standard deviation; IQR, interquartilic range range, NA, number of not available data; BMI, body mass index; Waist circ., waist circumference; SAP, systolic arterial pression; DAP, diastolic arterial pression; Creat, creatinine; eGFR, estimated glomerular filtration rate; CRP,C-reactive protein; ESR, Erythrocyte Sedimentation Rate; Tot.chol, total cholesterol; HDL.chol, High-density lipoprotein cholesterol; LDL. chol, Low-density lipoprotein; TG, Triglycerides; WBC, white blood cells count; neut.count, neutrophils; lymph.count, lymphocytes; mon.count, monocytes; MHR, monocyte to high-density lipoprotein cholesterol ratio; NLR, neutrophil to lymphocyte ratio; PLR, platelet to lymphocyte ratio; LMR, lymphocyte to monocyte ratio. (DOCX) [file pone.0231927.s002.docx]

**Table S2. Baseline characteristics of the population**

| **Variable** | **N** | **mean±SD**  **median±IQR** |
| --- | --- | --- |
| **Age** (years)* | 770 | 56.35±14.59 |
| **BMI (**kg/$m^{2}$**)*** | 771 | 27.98±5.93 |
| **Waist circ**. (cm)* | 771 | 99.34±15.11 |
| **SAP** (mmHg)* | 768 | 128.10±18.17 |
| **DAP** (mmHg)* | 768 | 78.80±10.31 |
| **Creat**( mg/dL)* | 765 | 0.82±0.19 |
| **eGFR** (mL/min)* | 763 | 91.77±18.08 |
| **Fasting Plasma Glucose** (mg/dL)** | 768 | 104.38[36.69] |
| **CRP** (mg/L)** | 462 | 2.90[0.1] |
| **ESR** (mm/H)** | 503 | 13.00[13.5] |
| **Tot.chol** (mg/dL)* | 771 | 182.5±43.42 |
| **HDL.chol** (mg/dL)* | 771 | 53.51±15.61 |
| **LDL. chol** (mg/dL)* | 771 | 103.70±34.56 |
| **TG** (mg/dL)** | 771 | 108.00[71.5] |
| **WBC** (x${10}^{3}/L$)* | 771 | 6.56±1.91 |
| **neut.count** (x${10}^{3}/L$)* | 771 | 3.85±1.43 |
| **lymph.count** (x${10}^{3}/L$)* | 771 | 2.07±0.73 |
| **mon.count** (x${10}^{3}/L$)* | 771 | 0.41±0.14 |
| **MHR *** | 771 | 8.57±4.45 |
| **NLR*** | 771 | 1.97±0.78 |
| **PLR*** | 771 | 125.47±47.42 |
| **LMR*** | 771 | 5.36±1.88 |
| **Gender** | | |
| **Male** | 771 | 391 (50.71) |
| **Female** |  | 380 (49.28) |
| **Smoke** | | |
| **Non smokers** | 761 | 503 (66.09) |
| **Smokers** |  | 258 (33.90) |
| **Body Mass Index** | | |
| **Normal weight** | 771 | 253 (32.81) |
| **Overweight** |  | 291 (37.74) |
| **Obesity** |  | 227 (29.44) |
| **Waist Circumference** | | |
| **<94 cm (males), <80 cm (females)** | 771 | 177 (22.95) |
| **>94 cm (males), >80 cm (females)** |  | 594 (77.04) |
| **Metabolic Syndrome** | | |
| **Controls** | 771 | 394 (51.10) |
| **Metabolic Syndrome** |  | 377 (48.89) |
| **Metabolic Syndrome criteria** | | |
| **0** | 771 | 89 (11.54) |
| **1** |  | 138 (17.89) |
| **2** |  | 167 (21.66) |
| **3** |  | 194 (25.16) |
| **4** |  | 126 (16.34) |
| **5** |  | 57 (7.39) |
| **Statin treatment** | | |
| **Not in treatment** | 769 | 615 (79.97) |
| **In treatment** |  | 154 (20.02) |
| **Other Lipid-Lowering Medication** | | |
| **Not in Treatment** | 771 | 737 (95.59) |
| **In treatment** |  | 34 (4.40) |

Values are expressed as mean ±Standard Deviation or median ± [IQR] respectively for normal (*) and non-normal (**) distributed numeric variables, and with n (%) for categorical ones. **Abbreviations: Number Obs.,** number of available data**; St.Dev,** standard deviation**; IQR,** interquartilic range range, **NA,** number of not available data**; BMI,** body mass index**; Waist circ**., waist circumference; **SAP,** systolic arterial pression; **DAP,** diastolic arterial pression; **Creat,** creatinine; **eGFR,** estimated glomerular filtration rate; **CRP**,C-reactive protein; **ESR,** Erythrocyte Sedimentation Rate; **Tot.chol,** total cholesterol; **HDL.chol,** High-density lipoprotein cholesterol; **LDL. chol,** Low-density lipoprotein; **TG**, Triglycerides; **WBC,** white blood cells count; **neut.count**, neutrophils; **lymph.count**, lymphocytes; **mon.count,** monocytes; **MHR,** monocyte to high-density lipoprotein cholesterol ratio; **NLR,** neutrophil to lymphocyte ratio; **PLR,** platelet to lymphocyte ratio; **LMR,** lymphocyte to monocyte ratio.
